# Metastatic Odyssey: Decoding the Genomic Journey from Primary Colorectal Cancer to Disseminated Disease

**DOI:** 10.3390/cancers18071062

**Published:** 2026-03-25

**Authors:** Taxiarchis Konstantinos Nikolouzakis, John Souglakos, Epameinondas Evangelos Kantidakis, Katerina Achilleos, Troye van Staden, Emmanuel Chrysos

**Affiliations:** 1 Department of General Surgery, University General Hospital of Heraklion, 71110 Heraklion, Greece; 2 Laboratory of Translational Oncology, Medical School, University of Crete, 70013 Heraklion, Greece; 3 Department of Medical Oncology, University General Hospital of Heraklion, 71110 Heraklion, Greece; 4 General Practice and Family Medicine, Venizeleion General Hospital, 71409 Heraklion, Greece; med11p1160114@med.uoc.gr; 5 Department of General Surgery, Wits School of Clinical Medicine, University of the Witwatersrand, 7 York Road, Parktown, Johannesburg 2193, South Africa

**Keywords:** colorectal cancer, metastasis, genomics, circulating tumor DNA, organotropism, tumor microenvironment

## Abstract

Metastatic colorectal cancer (mCRC) accounts for 90% of CRC-related mortality. This review synthesizes insights from comparative genomics tracing evolutionary trajectories from primary tumor to disseminated disease. Multi-region sequencing reveals metastatic seeding often occurs early—before clinical detection—challenging linear progression models. The metastatic bottleneck reduces clonal diversity while enriching for dissemination-competent traits including SMAD4 loss, PTEN inactivation and metabolic reprogramming. Organ-specific adaptation yields distinct molecular signatures: liver metastases exhibit Wnt hyperactivation and TGF-β-driven immune suppression; peritoneal tumors display mucinous features; brain metastases show HER2 enrichment. The immune microenvironment evolves toward immunosuppressive configurations, with Microsatellite instability high (MSI-H) tumors acquiring B2M or JAK1/2 mutations. Circulating tumor DNA (ctDNA) enables real-time tracking of clonal dynamics, detecting molecular residual disease months before radiographic progression. Therapeutic resistance follows predictable evolutionary trajectories—from RAS/BRAF mutations to EGFR ectodomain alterations, HER2/MET amplifications and lineage plasticity—with metastasis-specific mechanisms including microenvironmental protection and cellular dormancy. The clinical future lies in interception: leveraging liquid biopsies for early detection, targeting both tumor-intrinsic vulnerabilities and permissive metastatic niches and adapting therapy dynamically to anticipate resistance. Understanding this genomic odyssey is essential for transforming mCRC into a controllable chronic condition.

## Simple Summary

Colorectal cancer remains a leading cause of cancer-related mortality worldwide. Although early-stage disease is often curable, five-year survival declines to 13–18% once metastases develop, with nearly half of patients presenting with disseminated disease at diagnosis. Metastatic colorectal cancer is characterized by profound clinical, biological, and genetic heterogeneity, and therapeutic resistance remains a principal challenge. Understanding the timing and mechanisms of metastatic spread is therefore critical. This review synthesizes evidence on the genetic adaptations that enable tumor cells to colonize distant organs and explores how technologies such as liquid biopsy can track these evolutionary changes in real time. The goal is to shift from treating established metastases toward intercepting dissemination before it becomes clinically apparent, informed by an evolutionary framework.

## 1. Introduction

Colorectal cancer (CRC) remains a leading cause of cancer-related mortality worldwide, and the vast majority of these deaths are attributable to metastatic disease rather than the primary tumor itself [1]. When detected at a localized stage, CRC is often curable with surgery, with or without adjuvant systemic therapy. Once distant dissemination has occurred, however, the disease becomes systemic and, in most cases, no longer curable. The central clinical challenge is not simply tumor burden, but biological complexity. Metastatic lesions within the same patient frequently harbor distinct genomic alterations and may respond differently to therapy, limiting the effectiveness of single-target approaches and accelerating adaptive resistance [2]. This heterogeneity is not incidental. It reflects an evolutionary process shaped by continuous interactions between tumor cell genomes and the surrounding microenvironment. Metastasis should therefore not be viewed as a single late event, but as the outcome of dynamic selection operating over time. Historically, the Fearon–Vogelstein model framed colorectal tumorigenesis as a linear accumulation of genetic alterations (*APC* → *KRAS* → *TP53* and others), with metastasis positioned as the final step in that sequence [3,4]. While this framework remains foundational, advances in multi-region sequencing and phylogenetic analysis have complicated that picture. In many cases, metastatic spread follows branched evolutionary trajectories rather than a strictly linear path [5]. Several models now coexist to explain dissemination. Some patients appear to follow a relatively linear progression, such as primary tumor to liver and subsequently to lung. Others demonstrate parallel evolution, in which distinct subclones arising from a common ancestral population seed metastases independently. Polyclonal dissemination—where multiple genetically distinct clones contribute to metastatic deposits—has also been documented [6,7]. Increasingly, evidence suggests that early dissemination may occur while the primary tumor is still clinically undetectable, with metastatic founders showing only limited genomic divergence from the ancestral clone [8]. In contrast, late dissemination involves subclones that have undergone substantial diversification within the primary tumor before spreading. These patterns are not mutually exclusive. Within a single patient, metastases may have been seeded at different time points and through different evolutionary routes, creating a mosaic of lesions with distinct biological liabilities. At the molecular level, metastatic competence appears to arise from a combination of canonical driver alterations, chromosomal instability (CIN), and epigenetic plasticity. Although *APC*, *KRAS*, and *TP53* remain central to colorectal carcinogenesis, metastatic lesions are often enriched for alterations affecting pathways involved in cell motility, extracellular matrix interaction, and therapeutic resistance. Amplifications in *ERBB2* and *MET*, as well as mutations in *RNF43*, illustrate how Wnt signaling and receptor tyrosine kinase pathways may be reinforced during dissemination [9,10]. The Consensus Molecular Subtypes (CMS) further link tumor biology to metastatic behavior; the mesenchymal CMS4 subtype, characterized by TGF-β activation, stromal infiltration, and angiogenesis, is consistently associated with inferior outcomes and increased risk of distant relapse [11]. CIN, present in approximately 85% of CRCs, provides a substrate for ongoing diversification. By generating structural variation and copy number alterations, CIN expands the evolutionary search space available to tumor cell populations, facilitating adaptation to new selective pressures, including systemic therapy [12]. At the same time, non-genetic mechanisms contribute to metastatic potential. Stable epigenetic reprogramming toward stem-like or mesenchymal states can enhance invasiveness and stress tolerance without requiring additional permanent mutations [13]. The tumor microenvironment (TME) plays an integral role throughout this process. Within the primary site, hypoxia, inflammation, and immune surveillance select for clones capable of resisting apoptosis and evading immune detection. Disseminating cells must then survive in circulation, often by forming aggregates with platelets or other tumor cells, before successfully colonizing distant organs [14]. The concept of a pre-metastatic niche adds another layer of complexity: tumor-derived exosomes, cytokines, and bone marrow-derived cells can remodel distant tissues in advance of tumor cell arrival, lowering the threshold for successful implantation [15]. Once established at a secondary site, metastatic founder cells confront organ-specific constraints. The liver offers a nutrient-rich but immunologically active environment; the lung imposes distinct mechanical and immune pressures [16]. Adaptation to these niches drives further diversification within metastases themselves. The result is a pattern in which a relatively small founding population expands and evolves under local selective forces, generating both shared truncal mutations and extensive private genomic alterations across lesions. In this context, metastatic CRC (mCRC) can be viewed as the product of an evolutionary bottleneck followed by organ-specific diversification. The bottleneck restricts dissemination to a limited subset of clones with sufficient proliferative capacity, migratory potential, stress tolerance, and immune evasiveness. Subsequent expansion within metastatic sites introduces additional layers of heterogeneity shaped by the microenvironment and by therapeutic exposure [17,18]. Systemic therapy, rather than functioning purely as eradication, becomes another selective pressure—eliminating sensitive populations while permitting resistant clones to expand [19]. These evolutionary dynamics have important therapeutic implications. A static, single-target strategy is unlikely to achieve durable control in a system characterized by ongoing diversification and selection. Instead, an evolution-informed framework may be required—one that anticipates resistance, targets adaptive pathways involved in dissemination and niche remodeling, exploits vulnerabilities in CIN-high tumors, and considers adaptive dosing strategies aimed at maintaining competitive suppression of resistant subclones [19,20]. Although substantial progress has been made in characterizing the genomic landscape of colorectal cancer, the biological mechanisms that allow only a subset of tumors to disseminate and establish distant metastases remain incompletely understood. Increasing evidence suggests that metastatic competence may arise through a process of tumor evolution and clonal selection, in which genetically diverse tumor cell populations acquire the capacity to invade, disseminate and adapt to distant microenvironments [3,21,22]. Within this framework, dissemination may occur earlier in tumor development than previously assumed, while the eventual success of metastatic colonies depends on both intrinsic genomic alterations and the selective pressures imposed by the metastatic niche [14,23].

In the sections that follow, we examine the methodological advances that have enabled reconstruction of metastatic phylogenies, review the genomic and microenvironmental determinants of dissemination, and discuss how these insights can inform next-generation diagnostic and therapeutic strategies in metastatic CRC.

## 2. Methodological Revolution: Capturing Metastasis in Time and Space

### 2.1. Multi-Region Sequencing (MRS)

Single-site biopsies offer only a partial view of a tumor’s genomic landscape. In CRC, where spatial heterogeneity is common, this limitation becomes particularly consequential. MRS addresses this gap by performing bulk genomic profiling across multiple spatially distinct sectors of a primary tumor and its matched metastases. By comparing these regions, MRS enables reconstruction of subclonal architecture and clarifies how dissemination arises from specific evolutionary branches rather than from a uniform tumor mass [24,25]. Applied to CRC, MRS has reshaped the conceptual relationship between primary tumors and metastases. In a high-depth whole-exome sequencing study of spatially separated primary and metastatic samples, Wei and colleagues observed substantial variability between patients but comparatively constrained diversity within individual tumors [24]. In detail, their study demonstrated that all metastatic tumors in their cohort inherited multiple genetically distinct subclones from primary tumors, providing strong evidence for the existence of polyclonal seeding mechanisms in CRC metastasis while challenging monoclonal seeding models [24]. Notably, metastatic lesions frequently harbored multiple genetically distinct subclones originating from the primary tumor. This observation supports polyclonal seeding in CRC and challenges strictly monoclonal models of metastatic spread. Subclonal diversity, therefore, appears functionally permissive for metastatic competence.

Another consistent finding across MRS studies is that metastases tend to display reduced heterogeneity relative to their corresponding primaries [26]. This contraction of genomic diversity aligns with the evolutionary bottleneck hypothesis: only a subset of primary tumor clones possesses the phenotypic capacity to survive dissemination and colonization. Metastasis, in this framework, represents a strict selection of the most well adopted cells and not a random choice. Case-level analyses further underscore the clinical implications of spatial heterogeneity. Kogita et al. performed targeted resequencing of multiple primary colon tumors and associated metastases within individual patients [27]. In one case, four synchronous primary tumors exhibited entirely non-overlapping mutation profiles. The liver metastasis traced phylogenetically to only one of these primaries. Moreover, an *ERBB4* mutation present in the ancestral primary clone was absent in the metastatic lesion, illustrating clonal selection during dissemination and potential discordance between primary tumor genotyping and metastatic therapeutic targets. Such findings demonstrate how single-region sampling can misrepresent the genomic drivers relevant to systemic disease [27].

Technical Considerations: Bulk vs. Single-Cell Approaches:

Bulk MRS relies on deep sequencing of spatially distinct tissue blocks. It captures aggregate clonal signals and supports phylogenetic reconstruction with high sensitivity for subclonal variants, although rare cellular populations may remain obscured. Nonetheless, bulk MRS has provided the foundational phylogenetic framework for interpreting metastatic timing and seeding patterns [24,26]. Single-cell sequencing offers complementary resolution. By interrogating individual cells, it reveals rare clones, clonal hierarchies and cell-to-cell variation in genotype and transcriptional state. Platforms such as combined genome-and-transcriptome sequencing (e.g., G&T-Seq) enable parallel analysis of mutational profiles and gene expression programs associated with metastatic fitness [28]. An integrative atlas constructed from 35 single-cell RNA sequencing datasets and spatial transcriptomics samples from primary tumors and liver metastases identified a high-malignancy CRC subpopulation enriched in metastatic lesions [29]. These cells demonstrated stemness-associated programs, MYC-driven transcription and glycolytic reprogramming. Spatial mapping further localized them near cancer-associated fibroblasts at the tumor–stroma interface, suggesting metabolically organized niches that would not be evident in bulk data alone [29,30]. Integration of bulk and single-cell datasets—often through computational tools such as PyClone or Canopy—refines clonal inference and tracks expansion or contraction of specific subpopulations during metastasis or therapy [28]. Together, these approaches indicate that while single-region sequencing reliably captures truncal alterations, it systematically underestimates subclonal diversity that may underlie metastatic progression or therapeutic resistance [31].

### 2.2. Longitudinal Liquid Biopsies

Circulating tumor DNA (ctDNA) provides a non-invasive means of monitoring tumor evolution over time. Unlike resected specimens, which represent static snapshots, ctDNA reflects real-time tumor burden and clonal dynamics across the disease continuum—from primary resection to adjuvant therapy, relapse and subsequent treatment lines [32]. Serial ctDNA measurements can reveal shifts in clonal composition, detect minimal residual disease (MRD) and identify resistance-associated variants before radiographic progression becomes evident. In RAS-mutated metastatic CRC, ctDNA profiling has demonstrated utility for both prognostication and monitoring of treatment response. Rather than replacing tissue-based analysis, ctDNA extends it temporally, capturing evolutionary changes that occur under therapeutic pressure [33]. Two principal methodological strategies are used in ctDNA analysis. Tumor-informed assays begin with sequencing of the patient’s tumor tissue to identify somatic variants that are subsequently tracked in plasma. This personalized approach achieves high analytical sensitivity—variant allele frequency detection thresholds can reach 0.01–0.001%—and enables precise monitoring of known clonal markers. However, it cannot detect newly emergent mutations absent from the original tumor profile and requires adequate tissue for assay design [32]. Tumor-agnostic assays, by contrast, do not rely on prior tumor sequencing. Using predefined gene panels or broader genomic approaches, they interrogate ctDNA for mutations, structural variants and methylation signatures. Although typically less sensitive for individual low-frequency variants, they permit broader mutation discovery and are particularly useful when tumor tissue is unavailable [34]. The Guardant Reveal assay, evaluated in the UK TRACC Part B study, exemplifies this approach by combining somatic mutation detection with methylation profiling for tissue-free MRD assessment [35]. Technically, PCR-based methods such as digital droplet PCR offer high sensitivity for specific hotspot mutations, whereas next-generation sequencing-based platforms enable multiplexed detection of diverse genomic alterations. Error suppression techniques, including unique molecular identifiers and duplex sequencing, improve specificity by distinguishing true somatic variants from sequencing artifacts [33]. Comparative studies have clarified the performance characteristics of ctDNA relative to tissue sequencing. In a cohort of 84 patients with metastatic CRC receiving first-line therapy, Lee et al. compared tumor tissue DNA, baseline ctDNA and ctDNA at disease progression [36]. While tissue sequencing identified the highest total number of mutations, ctDNA analysis uncovered additional alterations in nearly half of patients. Post-progression ctDNA revealed novel pathogenic variants—absent from both baseline ctDNA and primary tissue—in approximately 12% of cases. These emergent alterations in *APC*, *TP53*, *SMAD4* and *CDH1* likely reflected clonal evolution under therapeutic selection. Quantitative ctDNA metrics also correlate with survival outcomes. Higher maximal VAF values at baseline were associated with inferior overall survival [36]. In a separate cohort, *KRAS* VAF greater than 20% corresponded to markedly reduced survival (12.1 months versus 42.9 months) [37]. These findings suggest that ctDNA burden functions not only as a molecular descriptor but also as a dynamic biomarker of disease aggressiveness. The TRACC Part B study prospectively evaluated longitudinal ctDNA monitoring in 214 patients with stage I–III CRC following resection [35]. Two-year recurrence-free survival was 91.1% among patients with undetectable postoperative ctDNA compared with 50.4% among those with detectable ctDNA. ctDNA positivity preceded radiologic recurrence by a median of 7.3 months, providing a potential window for early therapeutic intervention. Despite these advances, assay heterogeneity remains a challenge [34]. Differences in methodology, target selection and bioinformatic pipelines complicate cross-study comparisons and risk introducing variability unrelated to tumor biology. Standardization of positivity thresholds and analytical validation frameworks will be necessary before MRD-guided decision-making becomes routine clinical practice.

### 2.3. Metastatic Biopsy Programs & Autopsy Studies

Metastasis research has long been shaped by practical constraints. Clinically accessible lesions—most commonly in the liver and lung—are disproportionately represented in genomic studies, whereas metastases in the peritoneum, bone or ovary are often under-sampled [38]. This anatomical bias risks producing an incomplete representation of metastatic diversity. Structured metastatic biopsy programs attempt to address this imbalance by systematically sampling lesions from multiple organ sites. Such efforts allow comparative analysis of site-specific adaptation and inter-metastatic heterogeneity [7]. Rapid research autopsy programs extend this approach further. Conducted shortly after death, these protocols enable comprehensive collection of metastatic tissue across all involved organs, facilitating whole-body phylogenetic reconstruction [39]. Unlike clinically directed biopsies, rapid autopsies provide access to lesions that would otherwise remain uncharacterized. An early illustration of the value of complete metastatic mapping comes from Rübe et al., who analyzed 124 autopsy cases of colorectal carcinoma and correlated flow cytometric DNA content with patterns of metastatic spread [40]. It identified a subgroup of DNA-euploid primary tumors with distinctive biological behavior: these tumors exhibited an above-random frequency of liver-limited metastatic spread, with massive hepatic replacement often occurring in the absence of dissemination to lung or other distant sites. Importantly, their study also documented stemlines deviating in ploidy from the primary tumor in metastases from nine cases, providing early evidence for chromosomal heterogeneity and divergent evolution during metastatic progression [40]. They identified a subgroup of DNA-euploid primary tumors associated with liver-limited dissemination. Moreover, ploidy deviations between primaries and metastases in several cases suggested chromosomal divergence during progression. Such insights would have been inaccessible through selective biopsy of clinically dominant lesions alone. Modern autopsy programs now integrate next-generation sequencing, single-cell analysis and spatial transcriptomics. These datasets reveal substantial intra-patient heterogeneity across metastatic sites, including discordance in predictive biomarkers and emergence of resistance-associated variants [39]. Whole-body phylogenetic mapping has also uncovered inter-metastatic seeding and organ-specific evolutionary trajectories that are rarely detectable in limited biopsy cohorts [6,7].

## 3. The Genomic Archeology of Dissemination: When and How?

### 3.1. Timing of Spread: Early vs. Late Dissemination

**Figure 1:**
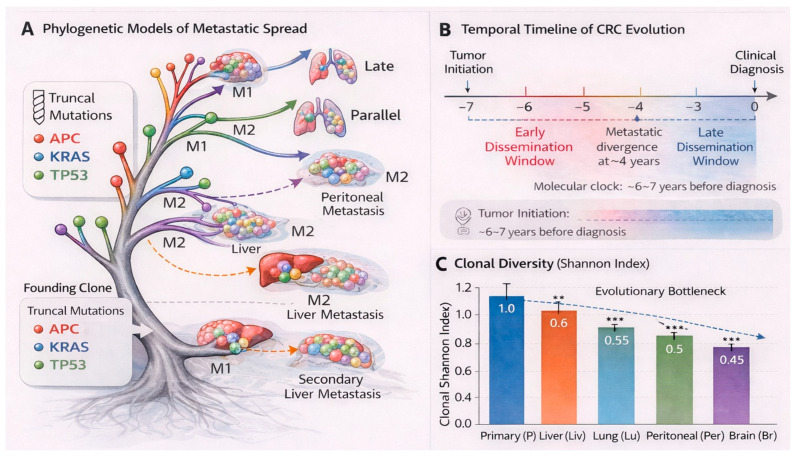
Evolutionary Trajectories and Timing of Metastatic Dissemination in Colorectal Cancer. (**A**) Phylogenetic models of metastatic spread based on multi-region sequencing studies. The founding clone harbors truncal mutations in *APC*, *KRAS*, and *TP53*. Subclonal diversification within the primary tumor gives rise to branches representing early dissemination (occurring when primary tumor is <0.01 cm^3^), late dissemination (after substantial primary diversification), parallel evolution (independent subclones seeding different metastases), and polyclonal seeding (multiple clones contributing to a single metastasis). Metastasis-to-metastasis seeding (arrow) illustrates secondary spread from established metastases. The evolutionary bottleneck reduces clonal diversity during dissemination. (**B**) Temporal timeline of CRC evolution based on molecular clock modeling. Tumor initiation occurs approximately 6–7 years before clinical diagnosis. Metastatic divergence occurs at ~4 years before diagnosis, with early dissemination (red window) and late dissemination (blue window) representing the windows during which seeding can occur. Micrometastases may be present at the time of primary resection in early-disseminated cases. (**C**) Clonal diversity (Shannon index) across anatomical sites, demonstrating the metastatic bottleneck. Primary tumors (P) show highest diversity (1.0), with progressive reduction in liver (Liv: 0.6), lung (Lu: 0.55), peritoneal (Per: 0.5), and brain (Br: 0.45) metastases. Error bars represent ±0.05. *** *p* < 0.01; ** *p* < 0.001 vs. primary tumor.

For many years, metastatic competence in CRC was conceptualized as a late-acquired trait. This view, rooted in the Fearon–Vogelstein model, assumed that dissemination followed the gradual accumulation of driver mutations within an advanced primary tumor [8]. Under this scope, metastasis represented the terminal stage of a linear genetic progression. More recent phylogenetic analyses, however, have complicated this narrative. Hu and colleagues performed exome sequencing on 118 biopsies from 23 patients with paired liver or brain metastases and applied a spatial tumor growth model to estimate patient-specific dissemination timing [8]. Their results suggested that metastatic seeding often occurs when the primary tumor is still clinically undetectable—frequently at volumes below 0.01 cm^3^. Among evaluable patients, 81% demonstrated evidence consistent with early dissemination. In many cases, metastatic founders diverged from the primary lineage long before diagnosis. Independent reconstruction by Alves et al. reached similar conclusions in a microsatellite-stable CRC case analyzed using multiregional [41]. Molecular clock modeling estimated tumor initiation approximately 6–7 years prior to diagnosis, with divergence of the metastatic ancestor occurring roughly 4 years before clinical detection. The interval between transformation and specification of metastatic competence appeared relatively narrow. Signals of positive selection were identified along the ancestral metastatic lineage, including a non-synonymous mutation in *ANGPT4*, an angiogenesis-related gene implicated in tumor progression [41]. Genomic similarity between primary tumors and metastases further supports early divergence in many cases. Hu et al. reported that approximately 70% of high-frequency somatic variants (cancer cell fraction > 60%) were shared between primaries and metastases [26,41]. Canonical drivers such as *APC*, *KRAS* and *TP53* were typically truncal, acquired early and retained across sites. By contrast, metastasis-private mutations were not enriched for classical driver genes, suggesting that once early dissemination has occurred, few additional canonical alterations are strictly required for metastatic outgrowth. These observations align with the “Big Bang” model of CRC evolution, in which substantial intratumoral heterogeneity arises early during tumor expansion and subsequent growth reflects largely neutral diversification among subclones of comparable fitness [8]. Under this model, metastatic competence may be embedded within early-arising lineages rather than emerging only after prolonged primary tumor evolution. That said, not all data conform to an early-dissemination paradigm. Leung and colleagues, using single-cell DNA sequencing, described cases in which metastatic lineages diverged only after substantial subclonal diversification within the primary tumor [7,8]. Similarly, Reiter et al. reported instances where metastases appeared to originate from relatively late-emerging primary subclones that had accumulated additional private mutations prior to dissemination [7]. These findings suggest that in some tumors, metastatic capability may indeed arise after extended intratumoral evolution. Part of the apparent discrepancy likely reflects methodological differences. Phylogenetic trees derived from bulk sequencing can give misleading impressions when interpreted without temporal calibration. Hu and colleagues emphasized that quantitative modeling—rather than visual inspection of branching structure alone—is required to infer dissemination timing reliably [8]. Low genomic divergence between primary and metastatic lesions, when analyzed within appropriate statistical frameworks, may indicate early seeding even if branching patterns appear complex. Importantly, early and late dissemination models need not be mutually exclusive. Population-level analyses suggest a continuum rather than a dichotomy. In the case described by Alves et al., early monoclonal seeding to the liver was followed by rapid expansion of both primary and metastatic populations, with metastatic growth slightly preceding primary clade expansion [41]. This pattern resembles punctuated dissemination—an early, discrete seeding event followed by parallel diversification at separate anatomical sites. Hu’s quantitative model likewise demonstrated variability across patients, with dissemination spanning a continuous spectrum from very early tumor growth to later primary stages [8]. The clinical implications are substantial. Patients with early-disseminated disease may harbor micrometastatic deposits at the time of primary resection, limiting the curative potential of surgery alone. Conversely, tumors in which dissemination occurs later may remain amenable to aggressive local strategies before systemic spread is fully established. Further complexity arises from polyclonal seeding and metastasis-to-metastasis spread. Alves et al. reconstructed a case in which an ancestral metastatic clone seeded the liver hematogenously, subsequently spread to hepatic lymph nodes and later appeared to reseed colonic lymph nodes—an example of retrograde or “self-seeding” dynamics [41]. Such patterns indicate that metastasis is not a single irreversible event but a dynamic and iterative process in which secondary lesions can themselves act as sources of further dissemination. Taken together, current evidence supports a heterogeneous temporal landscape of metastatic spread in CRC. Some tumors disseminate remarkably early, others later, and many may involve overlapping mechanisms. Rather than replacing the linear progression model outright, contemporary phylogenetic data suggest that metastatic timing varies across patients and may even differ among subclones within the same tumor. Figure 1 provides a visual representation of these evolutionary trajectories and timing models. Table 1 summarizes the principal evolutionary models—from linear progression to polyclonal and cross-metastatic seeding—along with their supporting genomic evidence and clinical implications.

### 3.2. Routes of Spread and Genomic Correlates

The anatomical route through which CRC disseminates is more complex than just a matter of vascular supply; it imposes distinct biological constraints that shape clonal selection and metastatic outgrowth. As comparative genomic datasets have expanded, it has become increasingly clear that metastases arising in different organs are not genomically interchangeable. Instead, they reflect site-specific adaptation layered upon shared ancestral mutations.

Portal Circulation (Liver): The liver is the most common site of CRC metastasis, largely owing to portal venous drainage from the colon and rectum. Yet anatomical access alone does not explain hepatic dominance. The liver microenvironment—rich in growth factors, metabolically active, and immunologically complex—appears to favor particular molecular configurations. Aberrant Wnt/β-catenin signaling has consistently emerged as a key feature of liver-tropic disease [46,47]. Single-cell transcriptomic profiling of colorectal liver metastases identified epithelial subpopulations enriched for Wnt/β-catenin and KRAS pathway activity [46]. These cells displayed recurrent copy number alterations, including loss of chromosomes 1 and 6p and gain of chromosomes 7 and 20q, suggesting that specific chromosomal architectures may enhance fitness within the hepatic niche. Reviews of this pathway have highlighted how canonical CRC driver alterations—*APC*, *CTNNB1* (β-catenin), and *KRAS*—converge to reinforce pathway hyperactivation in metastatic contexts [46]. Wnt ligands such as Wnt2, Wnt7b, Wnt3a and Wnt5a are upregulated within the metastatic microenvironment, establishing paracrine circuits that support stemness and proliferative persistence. Additional mechanisms appear to fine-tune colonization. Sciellin (SCEL), preferentially expressed in hepatic metastatic CRC cell lines, has been implicated in mesenchymal-to-epithelial transition (MET) through interaction with β-catenin and E-cadherin [47]. Its expression is modulated by TGF-β1 and hypoxia, pointing to a plastic and context-dependent regulatory axis. Functional studies using intrahepatic injection models demonstrated that SCEL expression is required for efficient metastatic growth in the liver, suggesting that hepatic colonization depends not only on invasive capacity but also on the ability to re-establish epithelial organization within the parenchyma [48]. Taken together, liver metastasis appears to require both canonical CRC driver signaling and organ-specific adaptations that enable survival in a metabolically abundant yet immunologically surveilled environment.

Lymphatic → Systemic (Lung): The relationship between lymphatic spread and subsequent hematogenous dissemination remains complex. Classical models proposed a sequential route—primary tumor to lymph nodes to distant organs. However, genomic reconstructions increasingly challenge this linear framework. Biogeographic phylogenetic analysis in a patient with extensive metastatic involvement suggested that liver metastases were seeded directly from the primary tumor via the portal circulation, whereas certain lymph node metastases appeared to arise secondarily from hepatic lesions [41]. This observation implies that lymph node metastases may, in some instances, represent offshoots of established distant metastases rather than obligatory intermediates. Metastases requiring systemic arterial dissemination, such as those to the brain, provide further insight into selective pressures beyond the liver. Comparative genomic analysis demonstrated that brain metastases harbored a greater number of private clonal mutations than liver metastases (median 24.5 versus 9.5) [8]. Whether this reflects later divergence, more stringent selective barriers—such as traversal of the blood–brain barrier—or accelerated post-colonization evolution remains uncertain. Amplification of *HTR2A*, encoding the serotonin receptor 2A, was observed more frequently in brain metastases than in liver lesions [8]. While mechanistic interpretation remains speculative, this finding suggests that successful colonization of distinct systemic sites may require adaptation to organ-specific signaling environments. Overall, systemic dissemination does not appear to follow a single obligatory path. Instead, metastatic spread likely involves parallel and sometimes recursive seeding events, with genomic diversification shaped by the selective landscapes of each organ.

Transcoelomic (Peritoneal): Peritoneal metastasis represents a fundamentally different mode of dissemination. Rather than intravascular transit, tumor cells breach the serosal surface, exfoliate into the peritoneal cavity, and implant onto mesothelial surfaces. Clinically, this route is associated with mucinous histology, *KRAS* mutations, and adverse prognosis. Loss or functional impairment of adhesion molecules—particularly E-cadherin (*CDH1*)—facilitates detachment from the primary tumor mass. Once free within the peritoneal cavity, cells must resist anoikis, adhere to mesothelial cells, invade the submesothelial stroma and secure vascularization in a frequently hypoxic and nutrient-variable environment. Although peritoneal metastases have not been as extensively genomically profiled as liver metastases, epidemiologic and molecular correlations offer clues. *BRAF V600E* mutations are strongly associated with peritoneal dissemination. In a cohort of 530 patients with metastatic CRC, peritoneal involvement was observed in 46% of *BRAF*-mutant tumors compared with 24% of *BRAF*-wild-type tumors [6,42,43,44]. This association was confirmed in a larger cohort of 1363 patients, in which *BRAF V600E* independently predicted peritoneal metastasis [45]. The clinicopathologic phenotype of *BRAF*-mutant CRC—right-sided origin, mucinous differentiation and infiltrative growth—may facilitate serosal penetration and exfoliation [44,49]. At a molecular level, transcriptional programs promoting epithelial–mesenchymal plasticity and features of the senescence-associated secretory phenotype (SASP) may remodel the tumor microenvironment toward a pro-inflammatory, stroma-rich state permissive for implantation [50,51]. Peritoneal dissemination, therefore, reflects not only physical proximity but also a molecular profile that supports survival outside the vascular compartment.

Although liver, lung and peritoneal metastases represent the dominant patterns of dissemination in colorectal cancer, several less common metastatic sites have been described and may reflect distinct biological pathways of tumor spread.

Ovarian Metastasis: Ovarian metastases from CRC represent a clinically challenging and biologically distinct manifestation of advanced disease. Median overall survival typically ranges from 10 to 30 months despite aggressive multimodality therapy [6,43]. Whole-exome sequencing of matched primary tumors, ovarian metastases and additional metastatic sites has revealed several distinctive features [6,42,43]. Mutations in genes such as *USP7* and *RPA1*—both linked to homologous recombination deficiency—have been proposed as candidate drivers or biomarkers in ovarian lesions. Ovarian metastases also demonstrate an “immune desert” phenotype, characterized by marked depletion of tumor-infiltrating lymphocytes relative to both primary tumors and other metastatic sites. Prognostically, patients segregate into subgroups based on mutational signature profiles. In one series, a high-risk subgroup defined by *USP7* mutation, increased copy number alterations and lower neoantigen burden exhibited shorter median survival compared with a more genomically stable subgroup [6,43]. Distinguishing metastatic CRC in the ovary from primary ovarian carcinoma remains critical. CRC-derived ovarian metastases lack the *BRCA1/2* mutation pattern typical of high-grade serous ovarian cancer and instead display mutational signatures consistent with colorectal origin [42,43]. This distinction has therapeutic implications, as CRC ovarian metastases respond poorly to platinum-based regimens that are effective in primary ovarian malignancies. Phylogenetic reconstruction suggests heterogeneity in routes of ovarian involvement. In some patients, ovarian metastases appear to derive directly from the primary tumor, consistent with transcoelomic or hematogenous spread. In others, ovarian lesions arise from pre-existing metastases, including peritoneal or lymph node sites [6,42,43]. Bilateral ovarian metastases within the same patient may even originate from different ancestral clones, underscoring the complexity of metastatic trafficking. CRC ovarian metastases thus exemplify organ-specific adaptive divergence. Once established within the ovarian microenvironment, metastatic clones acquire additional private alterations shaped by local selective pressures, further amplifying intra-patient heterogeneity [6,43].

Bone Metastasis: Although bone metastases are less common in CRC compared to primary lung or breast cancers, their incidence increases with prolonged survival and exposure to multiple lines of therapy [52]. Molecular analyses suggest that alterations affecting pathways involved in cell adhesion, extracellular matrix remodeling and PI3K–AKT signaling may contribute to bone tropism in colorectal cancer cells [53]. Skeletal dissemination is associated with specific genomic correlates: KRAS mutations, particularly in codon 12, have been linked to osteolytic bone lesions, while *TP53* alterations appear enriched in patients who develop bone involvement [54,55]. Clinically, bone metastases often present with pain, pathologic fractures, or spinal cord compression, and they carry a poor prognosis with median survival typically less than 12 months after detection [55,56].

Adrenal gland metastases: While often asymptomatic and discovered incidentally on surveillance imaging, occur in approximately 1–15% of patients with advanced CRC at autopsy [57,58]. Genomically, adrenal metastases have been associated with APC truncating mutations and, in some series, with *PIK3CA* alterations [9]. The adrenal microenvironment—rich in corticosteroids and catecholamines—may exert selective pressures that favor clones capable of surviving hormonal stress, though this remains speculative. Bilateral adrenal involvement can occasionally precipitate adrenal insufficiency, a medical emergency that requires prompt recognition and steroid replacement [57,58].

Brain metastases: Brain metastasis represent another uncommon but clinically challenging site, with *HER2* amplification emerging as a potential driver of intracranial colonization [59]. Of note, patients with isolated adrenal or bone metastases may occasionally be candidates for metastasectomy or stereotactic radiation, emphasizing the importance of recognizing these patterns [55,56]. However, the topic of CRC brain metastasis will be covered in greater detail in Section 4.2.

### 3.3. Epithelial–Mesenchymal Plasticity and Metastatic Competence

The acquisition of mesenchymal features—collectively termed epithelial–mesenchymal transition (EMT)—has long been recognized as a critical enabler of metastatic dissemination in CRC [60]. However, contemporary understanding has evolved from viewing EMT as a binary switch toward appreciating it as a dynamic, reversible spectrum of epithelial–mesenchymal plasticity (EMP) that allows tumor cells to adapt to changing microenvironmental demands throughout the metastatic cascade [61]. At the molecular level, EMT is orchestrated by a core set of transcription factors—SNAI1 (Snail), SNAI2 (Slug), ZEB1, ZEB2, TWIST1, and TWIST2—that coordinately repress epithelial genes (notably E-cadherin, encoded by *CDH1*) while activating mesenchymal programs including vimentin (VIM), fibronectin (FN1), and matrix metalloproteinases (MMPs) [62]. SNAI1 directly binds E-box elements in the *CDH1* promoter, recruiting chromatin modifiers such as histone deacetylases and polycomb repressive complexes to silence epithelial differentiation [63]. ZEB1 and ZEB2 function through similar mechanisms but also engage positive feedback loops that stabilize the mesenchymal state [64]. TWIST1, beyond its transcriptional functions, promotes cancer stem cell properties and metabolic reprogramming toward glycolysis—adaptations that may facilitate survival in nutrient-poor metastatic niches [65]. In CRC, EMT programs are activated by microenvironmental signals including TGF-β, Wnt, and inflammatory cytokines. TGF-β, abundantly present in the tumor microenvironment, induces SNAIL and ZEB expression through SMAD-dependent and independent pathways, linking stromal activation to tumor cell plasticity [66]. Notably, the CMS4 mesenchymal subtype—characterized by TGF-β activation, stromal infiltration, and poor prognosis—shows consistent upregulation of EMT transcription factors and corresponding downregulation of epithelial markers [67]. Importantly, complete EMT is rarely observed in human CRC metastases. Instead, circulating tumor cells and early metastatic deposits frequently exhibit partial or hybrid epithelial–mesenchymal phenotypes, co-expressing epithelial (EpCAM, cytokeratins) and mesenchymal (vimentin, fibronectin) markers [68]. This intermediate state may be functionally optimal: cells retaining some epithelial features maintain proliferative capacity, while partial mesenchymal characteristics confer migratory ability and stress resistance. Single-cell transcriptomic studies of CRC liver metastases have identified hybrid EMT states spatially localized at tumor-stroma interfaces, where cancer-associated fibroblasts (CAFs) provide juxtacrine and paracrine signals that sustain plasticity [29,69]. EMT programs also intersect with therapeutic resistance. Cells that have undergone partial EMT upregulate drug efflux pumps (ABCB1, ABCG2), anti-apoptotic proteins (BCL2, survivin), and DNA repair enzymes, rendering them less sensitive to conventional chemotherapy [69]. In the context of anti-EGFR therapy, ZEB1 expression has been associated with primary resistance even in *RAS*/*BRAF* wild-type tumors, suggesting that mesenchymal state—rather than specific mutations alone—may dictate treatment outcomes [69]. Conversely, reversion to an epithelial state (mesenchymal–epithelial transition, MET) appears necessary for metastatic colonization and outgrowth at secondary sites, highlighting the dynamic nature of this plasticity [70]. Therapeutically, targeting EMT directly remains challenging due to the absence of ‘druggable’ master regulators. However, strategies aimed at disrupting upstream signals—including TGF-β receptor inhibitors (galunisertib), FAK inhibitors (defactinib), and agents targeting CAF-derived factors—are being explored for their ability to suppress EMT-driven dissemination [71]. Given the central role of epithelial–mesenchymal plasticity in metastasis and resistance, understanding its regulation at single-cell resolution remains a priority for future research.

## 4. The Metastatic Bottleneck and Organ-Specific Adaptation

### 4.1. The Genomic Bottleneck: Selection, Not Chance

Across sequencing studies, one pattern recurs with striking consistency: metastatic lesions are usually less heterogeneous than their corresponding primary tumors. Primary colorectal cancers often resemble branching evolutionary trees with multiple coexisting subclones. By contrast, established metastases tend to look comparatively streamlined. Ham-Karim and colleagues examined 22 paired primary-metastasis samples and found that although mutation status was largely concordant overall, the internal heterogeneity patterns differed substantially between sites [72]. In other words, the same mutations were often present, but the clonal architecture had shifted. Certain subclones evident in the primary tumor were simply no longer represented in the metastasis. However, it is difficult to interpret this pattern as random sampling. If metastasis were purely stochastic—if any cell entering the circulation had an equal chance of founding a lesion—one would expect a broader representation of primary tumor diversity. Instead, what we repeatedly observe is contraction. Importantly, the bottleneck is not confined to the DNA sequence level. In the same study, widespread differences were observed in miRNA expression and protein biomarker profiles between primary and metastatic tumors [72]. The implication is that metastatic competence reflects coordinated molecular programs rather than isolated genetic accidents. This does not mean chance plays no role. Anatomical factors and vascular flow clearly influence where circulating tumor cells arrest. However, phylogenetic reconstructions across multiple cohorts indicate that metastases rarely arise from minor, apparently neutral subclones. They more commonly trace back to lineages already enriched for features consistent with invasion, stress tolerance, and immune evasion [31,73]. Even in cases of polyclonal seeding, where more than one subclone contributes to a metastasis, the overall diversity remains compressed relative to the primary tumor [25,59]. The pattern is one of selection from a restricted subset, not wholesale export of tumor complexity. At the level of recurrent drivers, the picture is familiar. *APC* and *TP53* alterations are typically truncal and shared across primary and metastatic compartments. In metastatic cohorts, *TP53* mutation rates often exceed 80%, with *APC* and *KRAS* close behind [73]. Li and colleagues, in a whole-exome sequencing study of 57 CRLM patients, similarly reported *APC* (64.91%), *TP53* (64.91%), *KRAS* (50.88%), *PIK3CA* (24.56%) and *SMAD4* (24.56%) as the most frequently mutated genes [74]. These early events likely establish the permissive background required for dissemination—Wnt pathway activation, genomic instability, resistance to apoptosis. Additional alterations appear enriched in metastatic branches. *SMAD4* loss, for example, disrupts canonical TGF-β signaling and may release cells from cytostatic control while preserving pro-invasive aspects of the pathway [75]. *PTEN* deletion enhances *PI3K–AKT* signaling and confers resistance to anoikis and metabolic stress [76]. Mutations in *FBXW7* stabilize multiple oncogenic substrates simultaneously. None of these alterations alone “cause” metastasis, but together they reinforce proliferative and survival capacity under hostile conditions. Whole-genome doubling is also frequently observed in metastatic lineages [77]. Polyploidization increases chromosomal instability and may provide a substrate for rapid adaptation once a metastatic colony is established. In that sense, the bottleneck does not simply restrict—it may set the stage for accelerated diversification after colonization. Taken together, the data favor a model in which metastasis reflects selective filtration of biologically equipped clones rather than a random founder effect.

### 4.2. Organ-Specific Genomic Adaptation

If the bottleneck determines which clones leave the primary site, the destination determines which ones persist. Each organ imposes its own constraints—metabolic, stromal, immunologic—and metastatic outgrowth appears shaped by those pressures.

Liver Metastases: The predominance of liver metastases in CRC is partly anatomical, but not entirely. Portal circulation explains access, not successful colonization. Genomic analyses of colorectal liver metastases (CRLM) consistently show reinforcement of Wnt signaling. *APC* mutations are common, and in some cases additional events further enhance β-catenin activity [73,74]. *SMAD4* inactivation, seen in a substantial minority of cases, may be particularly relevant in the TGF-β–rich hepatic environment [73]. Loss of p53 function is almost ubiquitous, underscoring its central role in tolerating dissemination-associated stress. Interestingly, an increasing amount of evidence explores metabolic adaptation. Transcriptional profiling has identified upregulation of genes such as *BCAT1* and *ALDH1L2* in liver metastases [73,78]. These alterations suggest that successful hepatic clones do not survive by chance but rather they reprogram metabolism to fit a microenvironment characterized by fluctuating oxygen levels and complex nutrient gradients. Prognostic associations further hint at site-specific biology. In one CRLM cohort, mutations in *ZNF717*, *POTEE*, and *MUC2* were associated with worse survival, whereas APC mutation correlated with improved outcomes [74]. These findings remind us that even canonical driver events can have context-dependent clinical effects.

Lung Metastases: Compared with liver lesions, pulmonary metastases remain less extensively characterized. Some reports suggest relative enrichment of *KRAS G12C* mutations in lung metastases [79], a potentially actionable observation given the development of *KRAS G12C* inhibitors. Whether this represents true biological selection or sampling bias remains uncertain. Transcriptomic studies have also described expression of NKX2-1 (TTF-1), a lung lineage–associated transcription factor, in subsets of CRC lung metastases [21]. This has been interpreted as possible lineage mimicry—an adaptive strategy whereby tumor cells engage organ-specific transcriptional programs to facilitate colonization. The pulmonary niche differs immunologically from the liver, with distinct patterns of T-cell and myeloid infiltration. The high-oxygen environment may favor clones capable of managing oxidative stress, although this remains speculative.

Peritoneal Metastases (Carcinomatosis): Peritoneal dissemination is biologically distinct from hematogenous spread. Here, tumor cells exfoliate into the peritoneal cavity and implant onto mesothelial surfaces. Clinically, this route is often associated with mucinous histology and *KRAS* mutation. Upregulation of mucin genes such as *MUC2* and *MUC5AC* contributes to the characteristic extracellular mucin seen in peritoneal metastases. Mucin production may provide mechanical and immunologic advantages, facilitating implantation and shielding tumor cells from host defenses. *CDH1* loss—through mutation or epigenetic silencing—further promotes detachment and plasticity [76], *BRAF V600E*–mutant CRC represents a relatively homogeneous subtype with a distinct metastatic pattern favoring peritoneal and nodal spread over liver involvement [45,80,81]. *BRAF* mutation is typically truncal, present across all compartments, indicating early specification. Subsequent evolution often includes *RNF43* co-mutation and whole-genome doubling events possibly reflecting adaptation to oncogene-induced stress [73,77].

**Figure 2:**
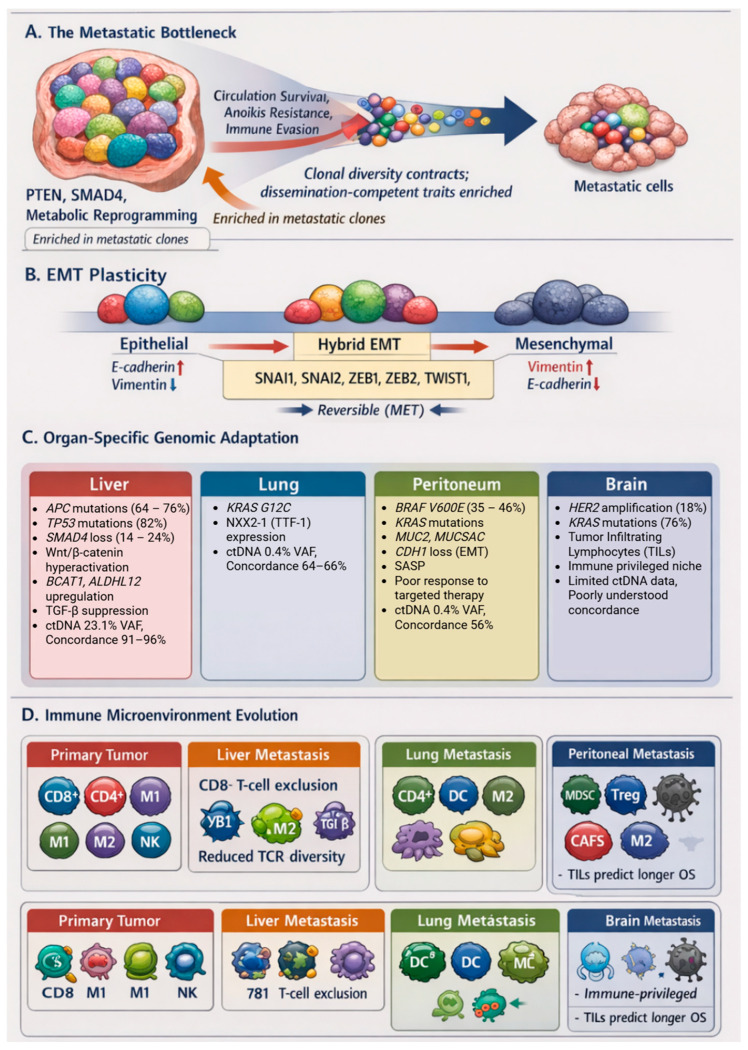
The Metastatic Bottleneck, Organ-Specific Adaptation, and Immune Microenvironment Remodeling. (**A**) The metastatic bottleneck restricts successful dissemination to a subset of primary tumor clones possessing traits required for survival in circulation, anoikis resistance, and immune evasion. Clonal diversity contracts while dissemination-competent traits (SMAD4 loss, PTEN inactivation, metabolic reprogramming) are enriched. (**B**) Epithelial–mesenchymal plasticity (EMP) enables adaptive responses throughout the cascade. Core transcription factors (SNAI1, SNAI2, ZEB1, ZEB2, TWIST1, TWIST2) orchestrate partial or hybrid EMT states, with complete mesenchymal transition rarely observed in established metastases. Mesenchymal–epithelial transition (MET) facilitates colonization at secondary sites. (**C**) Organ-specific genomic adaptations based on multi-region and liquid biopsy studies. Liver metastases exhibit APC (64–76%), TP53 (82%), SMAD4 loss (14–24%), Wnt hyperactivation, BCAT1/ALDH1L2 upregulation, TGF-β-driven immune suppression, and high ctDNA shedding (23.1% VAF, concordance 91–96%). Lung metastases show KRAS G12C enrichment, NKX2-1 expression, and low ctDNA shedding (0.4% VAF, concordance 64–66%). Peritoneal metastases display BRAF V600E enrichment (35–46%), KRAS mutations, MUC2/MUC5AC upregulation, CDH1 loss, SASP-driven inflammation, poor response to targeted therapy, and low ctDNA shedding (0.4% VAF, concordance 56%). Brain metastases demonstrate HER2 amplification (18%), KRAS mutations (76%), TILs, immune-privileged microenvironment, and limited ctDNA data. (**D**) Evolution of the immune microenvironment across metastatic sites. Primary tumors exhibit mixed immune infiltrates (CD8+, CD4+, M1, M2, NK). Liver metastases show CD8+ T-cell exclusion, Vδ1 T cells, reduced TCR diversity, and SPP1+ macrophages. Lung metastases display increased CD4+ T cells, dendritic cells, and M2-like macrophages. Peritoneal metastases exhibit marked enrichment of MDSCs, Tregs, CAFs, and M2 macrophages. Brain metastases show TILs that predict longer overall survival.

Brain Metastases: Brain metastases are rare in CRC but biologically intriguing. The HEROES study reported *HER2* amplification in a higher proportion of brain metastases than expected in unselected CRC populations [59]. This raises the possibility that *HER2* signaling may confer a selective advantage within the central nervous system. Interestingly, *KRAS* mutation in that cohort was associated with longer brain metastasis–specific progression-free survival illustrating that the prognostic impact of genomic alterations may vary depending on anatomical context. Lower Tumor mutation burden (TMB) (<5.02 mut/Mb) was associated with longer intracranial progression-free survival (HR 3.08; 95%CI 1.06–8.93; *p* = 0.0386) [59], suggesting that immunologically “cold” brain metastases with fewer neoantigens may evade intracranial immune surveillance more effectively. Adaptive changes enabling metabolic flexibility and immune evasion within the immune-privileged central nervous system are likely critical. Tumor-infiltrating lymphocytes (TILs) enriched brain metastases (≥1.6/HPF) conferred significantly longer overall survival (HR 0.11; 95%CI 0.01–0.91; *p* = 0.0403) [59], demonstrating the prognostic importance of the immune microenvironment even within the immune-privileged central nervous system. Beyond *HER2*, the HEROES study found no significant differences in mutation frequencies for *NRAS*, *BRAF*, MSI status, *TP53*, *APC*, or *PIK3CA* between primary tumors and brain metastases [59], suggesting that brain metastasis competence may be conferred by a limited set of specific alterations (particularly *HER2* amplification) superimposed on the standard CRC mutational landscape. The distinct genomic and immune features acquired during organ-specific adaptation are summarized in Table 2 and visualized in Figure 2, which integrates data on mutational signatures, microenvironmental characteristics, ctDNA shedding patterns and clinical implications for each major metastatic site.

## 5. The Evolutionary Dynamics of Treatment Resistance

### 5.1. Resistance in the Metastatic Setting

It is important to highlight that resistance rarely appears out of nowhere. In many patients, the seeds of resistance are already present—sometimes at vanishingly low frequencies—before treatment ever begins. What we often label as “acquired” resistance is, in practice, the selective expansion of pre-existing minor clones that were simply below the resolution of routine sequencing [86,87]. Ultra-deep sequencing of matched primary tumors and serial plasma samples has made this difficult to ignore. Resistant alleles can sometimes be detected months before radiographic progression becomes evident [86]. Under treatment pressure, these subclones expand rapidly. The tempo is often striking—mutant allele fractions can rise from undetectable to dominant within weeks. That pattern is far more consistent with strong selection than with slow, therapy-induced mutagenesis. The clearest example is anti-EGFR therapy in RAS wild-type metastatic colorectal cancer. In patients treated with cetuximab or panitumumab, resistant subclones harboring *KRAS*, *NRAS*, or *BRAF* mutations frequently emerge during therapy. Longitudinal ctDNA profiling has shown that many of these mutations were already present at very low levels prior to EGFR blockade [86]. Once the selective pressure is applied, they expand decisively. Clinically, this changes how resistance should be conceptualized. It suggests that deeper baseline sequencing—or highly sensitive plasma assays—might identify patients who are biologically predisposed to early resistance. It also supports the rationale for rational combinations upfront, rather than sequential monotherapies that allow one resistant lineage at a time to dominate. That said, as Pietrantonio et al. showed, the clonal architecture that unfolds under EGFR blockade can become remarkably complex, with both intra-lesion and inter-lesion diversity emerging over time [86,87]. In practice, this complexity limits how precisely we can intervene.

Resistance to BRAF/EGFR inhibition in *BRAF V600E*–mutant disease illustrates a parallel but distinct trajectory. Following treatment with encorafenib plus cetuximab, as established in the BEACON CRC pathway [25,88,89,90], ctDNA analyses have documented acquired *KRAS* amplification, *NRAS* mutations, *BRAF* amplification, and *MEK1* mutations as dominant escape routes [86,87]. These alterations converge on MAPK reactivation, but the specific genetic solutions vary from patient to patient. Importantly, multiple resistance mechanisms can coexist simultaneously. Different metastatic lesions within the same individual may harbor distinct alterations—a phenomenon of inter-lesion heterogeneity that makes single-site biopsy inherently incomplete [86]. Plasma-based monitoring offers a broader composite view, though even that has limitations. In *BRAF*-mutant patients, the high prevalence of peritoneal metastases—lesions that shed relatively little ctDNA—can blunt the sensitivity of liquid biopsy approaches [84,85]. The earliest resistance mechanisms are typically straightforward in principle: mutations in *KRAS*, *NRAS*, or *BRAF* reactivate downstream MAPK signaling and render upstream receptor blockade ineffective [86]. MET amplification can also emerge early and is associated with particularly poor outcomes under EGFR inhibition [91]. With prolonged exposure, more structurally elegant adaptations appear, such as extracellular domain mutations in EGFR (most commonly S492R) that prevent antibody binding while preserving receptor function [86,87]. At that stage, the tumor is no longer dependent on the original driver. The identification of MET amplification is clinically meaningful because it introduces a new therapeutic vulnerability, at least in principle. At the far end of the adaptive spectrum lies lineage plasticity. In rare but increasingly recognized cases, colorectal adenocarcinoma can undergo neuroendocrine transdifferentiation, acquiring small-cell morphology and corresponding transcriptional programs [92,93]. His represents not just pathway reactivation but wholesale phenotypic reprogramming. The molecular triggers remain incompletely defined, though epigenetic remodeling and stem-like plasticity almost certainly play a role. Clinically, these transformations are aggressive and broadly treatment-refractory. What becomes clear across these examples is that resistance in metastatic CRC is rarely linear. It is branching, parallel, and dynamic. Multiple clones compete under systemic pressure, and dominance shifts as the selective landscape changes [92].

### 5.2. Metastasis-Specific Resistance Mechanisms

Not all resistance is encoded in the tumor genome. The metastatic niche itself can actively shield tumor cells from therapy. This is especially evident in the liver. The hepatic microenvironment is not a passive bystander. Hepatic stellate cells, cancer-associated fibroblasts, and other stromal elements secrete factors that support tumor cell survival even when systemic therapy appears effective elsewhere [94,95]. Hepatocyte growth factor (HGF) is a particularly important mediator. Secreted by stromal cells, HGF activates MET signaling in adjacent tumor cells and can blunt the efficacy of EGFR inhibition, even in the absence of MET amplification [96,97]. Bradley et al. demonstrated that co-culture of colorectal cancer cells with HGF-producing fibroblasts induces migration, invasion, and resistance to MEK inhibition—a phenotype that could be attenuated with HGF-neutralizing antibodies [96]. This is not resistance in the classical, tumor-cell-autonomous sense. It is resistance as a product of bidirectional signaling between cancer cells and host tissue. There is also a metabolic dimension. Under conditions mimicking anti-angiogenic therapy, tumor cells appear more vulnerable to nutrient deprivation than to hypoxia per se. Stroma-derived HGF can protect against starvation-induced apoptosis by enhancing GLUT1-mediated glucose uptake and promoting autophagy [98]. This metabolic adaptation allows tumor cells to persist in environments where angiogenesis inhibition has compromised nutrient delivery. Combined targeting of HGF and metabolic pathways has shown preclinical promise without exacerbating hypoxia [99] suggesting that microenvironment-directed strategies may be necessary in certain contexts. Cytokines such as IL-6 and TGF-β within hepatic and peritoneal niches further promote survival and epithelial plasticity [14]. Resistance often reflects a dynamic ecosystem rather than a single mutational event. Immune resistance is also strongly site-dependent. Liver metastases, in particular, appear to attenuate responses to immunotherapy even in microsatellite instability–high disease [95]. The hepatic immune milieu is enriched in regulatory T cells, myeloid-derived suppressor cells, and immunosuppressive cytokines. Novel strategies targeting LAG-3 or VEGFR in combination with PD-1/PD-L1 blockade are being explored to overcome this barrier [95]. Even when therapy achieves apparent complete remission, disseminated tumor cells (DTCs) can persist in a state of quiescent dormancy for years or decades before eventually reactivating to produce overt metastases. This phenomenon, comprehensively reviewed by Chen et al., represents one of the most clinically significant yet poorly understood aspects of metastatic biology [94]. These cells are not actively proliferating, which makes them inherently resistant to cycle-dependent chemotherapy. Dormancy is regulated by a balance of stress-response pathways, including NR2F1 and p38 MAPK signaling [100]. High p38 and low ERK activity favor quiescence; reversal of that balance permits re-entry into proliferation. Dormant cells often rely more on epigenetic and transcriptional programs than on continuous mutational evolution [101]. When reactivation occurs—whether triggered by inflammation, stromal remodeling, or loss of suppressive cues—the resulting metastasis may display features distinct from both the primary tumor and earlier lesions [102]. Understanding how to either maintain permanent dormancy or eliminate dormant cells preemptively represents one of the major unmet challenges in metastatic oncology [103]. An especially intriguing illustration of evolutionary dynamics is the so-called “Lazarus” response. After discontinuation of anti-EGFR therapy, RAS-mutant resistant clones can decline in frequency, and ctDNA may revert to an apparently wild-type profile [104]. This likely reflects a fitness cost associated with resistance mutations in the absence of drug pressure [105,106]. In selected patients, rechallenge with anti-EGFR therapy can restore temporary sensitivity. In rare cases, true genetic reversion events have even been documented [80]. More commonly, however, it is a matter of shifting clonal dominance. When selective pressure is removed, fitter drug-sensitive clones re-expand. This oscillation between sensitive and resistant states suggests that continuous maximal suppression may not always be evolutionarily optimal. Adaptive treatment strategies that exploit fitness trade-offs may, in theory, prolong disease control [79,86].

## 6. Circulating Tumor DNA (ctDNA) as a Window into Metastatic Biology

### 6.1. Minimal Residual Disease Detection

Among all translational applications of ctDNA, postoperative detection of MRD has demonstrated the strongest prognostic signal in colorectal cancer. In patients undergoing curative-intent resection of primary tumors or oligometastatic lesions, detectable ctDNA in the postoperative setting is a near-binary marker of occult systemic dissemination. A systematic review and meta-analysis by Negro and colleagues, encompassing seven studies of stage II colorectal cancer, demonstrated that postoperative ctDNA positivity significantly increases the risk of recurrence, with a pooled risk ratio of 3.66 (95% confidence interval [CI]: 1.25–10.72; *p* = 0.002) [107]. This effect size positions ctDNA as the most powerful postoperative prognostic biomarker identified, surpassing traditional clinicopathologic risk factors including T-stage, lymphovascular invasion and even postoperative carcinoembryonic antigen (CEA) levels [107]. Numerous prospective cohorts have shown that ctDNA positivity following surgery is associated with recurrence rates exceeding 70–80%, compared to rates below 10–15% in ctDNA-negative individuals [107,108]. Cohen and colleagues’ real-world data analysis of 795 patients with stage I-III colon cancer, comprising 5971 serial plasma samples, further validated these findings [108]. CtDNA-positive patients during the MRD window showed dramatically shorter disease-free survival compared to ctDNA-negative patients (hazard ratio [HR]: 9.85, *p* < 0.0001). On multivariate analysis, ctDNA positivity emerged as the most significant factor associated with inferior DFS (adjusted HR: 7.7, *p* < 0.001), independent of stage, grade and adjuvant treatment exposure [108]. In this context, ctDNA functions not just as a biomarker of tumor burden but rather as a direct molecular readout of persisting micrometastatic clones that have survived surgical extirpation. Critically, ctDNA status also predicts benefit from adjuvant chemotherapy. MRD-positive patients who received adjuvant treatment showed improved DFS compared to those observed post-surgery (adjusted HR: 6.1, false discovery rate-adjusted *p* = 0.0007), whereas no chemotherapy benefit was observed in MRD-negative patients (adjusted HR: 1.20, *p* = 0.768) [108]. These findings provide the rationale for ctDNA-guided therapeutic decision-making, enabling treatment escalation for patients with residual disease and de-escalation for those likely already cured. Importantly, ctDNA detection precedes radiographic recurrence by a substantial margin. Yu et al., in their review of postoperative surveillance strategies, reported that ctDNA provides lead times of 3–11 months over conventional imaging and CEA monitoring [109]. Sasaki and colleagues, using digital PCR-based ctDNA monitoring with patient-specific mutation panels, documented a median lead time of 182 days (range, 0–376 days) from molecular relapse to clinical relapse [110]. The lead time between molecular relapse and imaging-confirmed progression effectively redefines the window of therapeutic intervention. This interval provides an opportunity for earlier systemic therapy, enrollment into MRD-directed clinical trials, or escalation strategies tailored to high-risk molecular profiles. The implications are profound: recurrence is no longer defined solely by macroscopic disease, but by the molecular reappearance of malignant clones in circulation. If computed tomography surveillance were extended to annual intervals and guided by ctDNA monitoring, Sasaki and colleagues estimated that 57.1% of CT scans could be avoided while still detecting all first clinical relapses [110]. Moreover, ctDNA monitoring would have provided a lead time of 339 days for detection of clinical relapse (range, 42–533 days) in a scenario where annual CT was supplemented by ctDNA surveillance [110]. This integrated approach—combining ctDNA’s temporal “signal” with imaging’s spatial “localization”—promises to accelerate detection of oligometastatic relapse while reducing unnecessary radiation exposure and healthcare costs [109]. Apart from that, ctDNA enables reconstruction of clonal phylogeny directly from plasma. By tracking variant allele frequencies and co-occurring mutations longitudinally, it is possible to infer the relative contribution of truncal versus subclonal populations, to identify emerging metastatic lineages and to determine whether recurrence arises from a single dominant clone or multiple independent expansions. Nagayama and colleagues provided insights into the biological significance of ctDNA-detectable clones, demonstrating that mutated genes identified in ctDNA from patients with postoperative recurrence exhibited immune-resistance traits in the primary tumor [111]. Compared with all mutated genes in primary tumors, the neoantigen peptides from ctDNA-detected mutations showed abundant expression but reduced binding affinity to HLA, suggesting that these clones had escaped immune surveillance through impaired neoantigen presentation [111]. This finding reveals that ctDNA not only identifies residual disease but also reports on the immune-evasive properties of metastatic clones. In certain cases, plasma-based phylogenetic inference has revealed polyclonal metastatic seeding that was not apparent from tissue sampling alone. Thus, ctDNA does not simply predict relapse—it allows reconstruction of the metastatic genealogy without repeated invasive biopsies. Ganesan and colleagues evaluated Bridge Capture technology, a novel next-generation sequencing-based platform for comprehensive ctDNA profiling, demonstrating strong correlation with droplet digital PCR for VAF values (rs = 0.86) and substantial concordance (kappa = 0.70–0.79) [112]. Importantly, Bridge Capture identified several oncogenic mutations beyond those detected by targeted hotspot panels, highlighting its capability for comprehensive profiling that could inform phylogenetic reconstruction [112].

### 6.2. Metastatic Heterogeneity Captured in Blood

The metastatic process in colorectal cancer is inherently heterogeneous, characterized by spatial divergence between primary tumors and distinct organ-specific metastases. Tissue biopsies, while indispensable, are inherently limited by sampling bias. A core needle biopsy from a single hepatic lesion may fail to capture subclonal populations residing in other liver segments, lung nodules, or peritoneal implants. ctDNA, by contrast, integrates genomic material shed from multiple metastatic deposits, thereby providing a composite representation of systemic clonal architecture. Wang and colleagues evaluated 76 CRC patients with paired tissue and plasma samples, identifying 26 cancer-related genes with most common variants in *APC* (57.9% tissue vs. 19.7% plasma), *TP53* (55.3% vs. 22.4%) and *KRAS* (47.4% vs. 43.4%) [113]. Overall concordance for actionable gene mutations was 73.53%. Notably, plasma ctDNA improved detection for certain genes and gene pools and combining tissue and plasma increased TMB detection [113]. Factors significantly associated with discordance included gender and the presence of peritoneal metastases, suggesting that clinical variables influence ctDNA shed and should be considered when interpreting liquid biopsy results. Concordance between ctDNA and matched tissue sequencing for canonical driver alterations—such as mutations in *KRAS*, *NRAS*, *BRAF*, *TP53* and *APC*—typically ranges from approximately 85% to 90% [84,113]. Manca and colleagues, in the Valentino study of 120 patients with left-sided, *RAS*/*BRAF* wild-type, *HER2*-negative mCRC, reported positive concordance between tissue and ctDNA of only 31.3% for RAS mutations and 47.1% for PIK3CA mutations [114]. However, presence of these mutations in baseline ctDNA was strongly associated with worse outcomes: median progression-free survival (PFS) of 8 versus 12.8 months for *RAS* mutations (HR 2.49; 95% CI 1.28–4.81; *p* = 0.007) and 8.5 versus 12.9 months for *PIK3CA* mutations (HR 2.86; 95% CI 1.63–5.04; *p* < 0.001). Patients with higher VAF levels (≥5%) had the worst outcomes (median PFS 7.7 vs. 13.1 months; HR 4.02; 95% CI 2.03–7.95; *p* < 0.001) [114]. These findings indicate that even when ctDNA fails to detect mutations present in tissue, its detection provides independent prognostic information reflecting tumor biology and burden. This high degree of overlap confirms the analytical validity of plasma-based genotyping for actionable alterations. However, ctDNA frequently reveals additional subclonal variants not detected in single-lesion biopsies, particularly those associated with emerging resistance mechanisms. Lee and colleagues performed targeted sequencing of 88 cancer-associated genes comparing tumor tissue DNA, baseline ctDNA and ctDNA obtained at progressive disease in 84 patients with metastatic CRC receiving first-line systemic therapy [36]. Sequencing ctDNA alongside tissue DNA revealed additional mutations in 40 patients (47.6%) and post-treatment ctDNA analysis unveiled 13 novel pathogenic mutations in 10 patients (11.9%) that were absent from both primary tissue and baseline ctDNA [36]. In this way, ctDNA often provides a more comprehensive and evolutionarily informative profile than tissue alone. Nevertheless, ctDNA analysis is not without biological constraints. The degree of tumor DNA shedding into circulation varies significantly according to metastatic site and tumor burden. Liver metastases, owing to their vascularity and direct drainage into the systemic circulation, are associated with high levels of ctDNA release and robust detection sensitivity. Kagawa and colleagues, in the METABEAM study of 221 patients with single-site metastases, reported concordance rates of 91% for liver metastases, 88% for peritoneal metastases and 64% for lung metastases [84]. Factors associated with concordance included baseline longest diameter and lesion number (*p* = 0.004) and sample collection interval (*p* = 0.036). Concordance rates ≥ 90% were observed in liver metastases regardless of tumor burden, peritoneal metastases with baseline diameter ≥ 20 mm and lung metastases with diameter ≥ 20 mm and/or lesion number ≥ 10 [84]. Bando and colleagues from the SCRUM-Japan GOZILA study specifically investigated the relationship between metastatic site and ctDNA detection in 138 patients with single-organ metastases [85]. Concordance of RAS/BRAF status between ctDNA and tissue was 95.9% in patients with liver-only metastases, but only 80.0% in lymph node-only, 56.0% in peritoneum-only and 65.9% in lung-only metastases. ctDNA fraction, measured by median maximum VAF, varied dramatically by site: 23.1% in liver-only, 6.0% in lymph node-only, 0.4% in peritoneum-only and 0.4% in lung-only metastases (*p* < 0.001). Few patients with liver-only (2.0%) or lymph node-only metastases (13.3%) had maximum VAF < 0.2%, the threshold required to ensure 95% detection sensitivity for subclonal variants. In contrast, maximum VAF was <0.2% in 27.7% of patients with lung-only and 29.6% with peritoneum-only metastases [85]. The biological basis for these differences likely reflects the vascularity and tissue architecture of each metastatic site. Liver metastases, with their rich sinusoidal blood supply and direct contact with the circulation, shed DNA efficiently into the bloodstream. Lung metastases, while highly vascular, may release DNA that is filtered or diluted before reaching peripheral circulation. Peritoneal metastases, existing as suspended aggregates or implants on serosal surfaces, may shed DNA directly into the peritoneal cavity rather than the bloodstream, limiting systemic detection [115]. These findings have critical clinical implications. For patients with lung-only or peritoneum-only metastatic disease, a negative ctDNA result does not reliably exclude the presence of clinically significant metastases or resistance mutations. Such patients may benefit from concurrent tissue and plasma testing to provide optimal genotyping for subsequent therapy selection [85,115]. Conversely, for patients with liver metastases, ctDNA provides a highly sensitive and comprehensive window into metastatic genomics, often surpassing tissue biopsy in capturing clonal diversity.

### 6.3. Cell Free RNA (cfRNA)

Beyond DNA-based approaches, emerging evidence supports the utility of cell-free RNA (cfRNA) profiling as a complementary liquid biopsy modality in colorectal cancer. Unlike ctDNA, which provides static genomic information, cfRNA captures real-time transcriptional dynamics, including gene expression programs, alternative splicing events, and non-coding RNA signatures that reflect active tumor biology and microenvironmental interactions [116]. Circulating tumor-derived RNA is often encapsulated within extracellular vesicles (exosomes) or bound to ribonucleoprotein complexes, which protect it from degradation and enable detection of tissue-specific transcripts [117]. In CRC, cfRNA profiling has shown promise for both early detection and monitoring. Plasma levels of exosomal mRNAs encoding epithelial–mesenchymal transition (EMT) regulators—including VIM, ZEB1, and TWIST1—have been correlated with metastatic burden and treatment response [67]. MicroRNA (miRNA) signatures, particularly panels incorporating miR-21, miR-29a, and miR-92a, have demonstrated diagnostic accuracy for primary CRC and may predict relapse following curative-intent surgery [118]. Long non-coding RNAs such as CCAT1 and HOTAIR, detectable in plasma, have been associated with liver metastasis and poor prognosis [119,120]. Importantly, cfRNA may address certain limitations of ctDNA analysis. Whereas ctDNA detection depends on the presence of tumor-derived somatic mutations—which may be absent in some patients—cfRNA signals can be derived from epigenetic and transcriptional alterations common across most CRC subtypes [121]. Moreover, cfRNA can provide insights into tumor plasticity and lineage switching that are not captured by genomic profiling alone. For example, emergence of neuroendocrine features during treatment resistance—a phenomenon discussed in Section 5.1—may be detected earlier through cfRNA monitoring of chromogranin A or synaptophysin transcripts than through standard imaging or ctDNA [122]. The integration of cfRNA with ctDNA analysis—sometimes termed ‘liquid biopsy multi-omics’—represents an evolving frontier. Combined DNA and RNA profiling from a single blood draw could simultaneously track clonal evolution (via ctDNA) and functional adaptation (via cfRNA), offering a more complete picture of metastatic dynamics [123]. Several groups are now exploring whether cfRNA methylation patterns can complement ctDNA for MRD detection, potentially overcoming the sensitivity limitations imposed by low ctDNA shedding in patients with peritoneal or lung-only metastases [85]. While cfRNA assays are less standardized than ctDNA methods and require careful pre-analytical handling to minimize RNA degradation, their biological richness justifies continued investigation.

## 7. The Immune Genomics of Metastasis

### 7.1. Evolution of the Immune Microenvironment Across Metastatic Sites

The immune landscape of CRC does not remain static as the disease progresses. It changes—sometimes subtly, sometimes dramatically—as tumor cells move from the colon to distant organs. The framework of immunoediting (elimination, equilibrium, escape) remains useful, but in metastatic disease the process feels less linear and more spatially fragmented [79,124]. Primary tumors can be immunologically diverse. Some are heavily infiltrated by cytotoxic CD8+ T cells and show features of active immune engagement. Others are excluded or frankly “cold.” What becomes apparent when matched metastases are examined is that these immune architectures do not simply replicate themselves elsewhere. Instead, metastatic lesions often diverge from their primaries, reflecting both clonal selection and the influence of organ-specific immune environments [79,125]. Macrophage polarization also shifts. While primary tumors often contain a mix of M1- and M2-like populations, liver metastases are commonly enriched in M2-polarized macrophages, which support tumor growth and suppress cytotoxic T-cell activity [82,125]. Checkpoint molecule expression, including PD-L1, may increase in metastatic sites, suggesting an adaptive response to immune pressure rather than baseline expression alone [126,127]. The cumulative effect is an immune context that is less inflammatory and more permissive. Luo et al. examined this question at scale, analyzing hundreds of primary and metastatic samples with integrated CMS and tumor microenvironment profiling [125]. Approximately two-thirds of metastases retained the same CMS subtype as their matched primaries, but the immune–stromal composition varied substantially by site. Liver metastases were predominantly CMS2, the more epithelial subtype, and showed comparatively reduced fibroblast infiltration. Lung and peritoneal metastases were more frequently CMS4—the mesenchymal subtype—accompanied by greater stromal and macrophage enrichment [125]. These site-specific immune configurations are better understood under the lens of the immunogenomic substrate: tumor cells of different CMS groups display preferential metastasis to organs whose immune-microenvironmental background proves permissive for their particular biological characteristics [125]. The mesenchymal CMS4 tumors, with their inherent TGF-β activation and stromal interaction programs, appear better adapted to the peritoneal and pulmonary microenvironments, while epithelial CMS2 tumors preferentially colonize the liver [128]. Whether this reflects intrinsic compatibility or selective survival after arrival is still debated, but the association is consistent. Across sites, however, a common theme emerges: metastases are often immunologically colder than their primaries. Increased regulatory T cells, expansion of myeloid-derived suppressor cells, and reduced cytotoxic T-cell density are recurrent findings—especially in the liver [82,129,130]. Tumor-intrinsic signaling pathways contribute to this shift. Activation of β-catenin, common in colorectal metastases, has been linked to impaired dendritic cell recruitment and T-cell exclusion, providing a direct connection between oncogenic signaling and immune escape [131]. The hepatic microenvironment deserves particular attention. Henriques et al. demonstrated in preclinical models and patient-derived data that TGF-β plays a central role in shaping the immune barrier in liver metastases [82]. TGF-β simultaneously limits recruitment of peripheral memory CD8+ T cells and instructs tumor-associated macrophages to suppress the clonal expansion of those that do arrive. Through induction of SPP1 (osteopontin), macrophages promote collagen deposition and fibroblast accumulation, further reinforcing immune exclusion. The result is more than reduced infiltration, since structural and functional insulation from immune attack are greatly involved. Cancer cells themselves participate actively in constructing this environment. Dang et al. identified cathepsin C (CTSC) as a driver of metastatic progression through enhanced recruitment of MDSCs and TAMs via the CSF1/CSF1R axis [82,130]. Inhibition of CTSC, particularly when combined with PD-L1 blockade, curtailed metastatic growth in preclinical models. Similarly, Chen et al. showed that tumor-intrinsic TGF-β signaling can directly induce PD-L1 expression, while CSF1-mediated macrophage recruitment sustains this state in a self-reinforcing loop [126,130]. Chemorefractory cancer cell-derived CSF1 then recruits additional TAMs, which sustain TGF-β-mediated PD-L1 expression in a vicious cycle. High macrophage infiltration correlated clinically with tumor PD-L1 status after chemotherapy in CRC patients. Depletion of immunosuppressive CSF1R^+^ TAMs combined with TGF-β receptor blockade resulted in increased influx of cytotoxic CD8+ T cells and effector memory CD8+ cells, reduction in Tregs and synergistic inhibition of tumor growth when combined with chemotherapy [126]. These findings identify CSF1R^+^ TAMs and TGF-β as dominant components regulating PD-L1 expression within the immunosuppressive metastatic microenvironment. Beyond the general trend toward immunosuppression, each metastatic site exhibits distinctive immune characteristics with therapeutic implications. Liver metastases are notable for their unique composition of γδ T cells. Bruni and colleagues demonstrated that the microenvironment of colorectal liver metastases is enriched for Vδ1 T cells, a subset of tissue-resident γδ T lymphocytes with high antitumor potential and natural tropism for the liver [83]. These Vδ1 T cells, particularly those expressing CD69 and exhibiting a terminally differentiated effector memory (TEMRA) phenotype, correlated with better clinical outcomes including fewer metastatic lesions and longer overall survival. Importantly, these intrahepatic CD69^+^ Vδ1 T cells could egress from the liver and re-circulate in peripheral blood, where their frequencies maintained prognostic significance independent of neoadjuvant therapy [83]. This finding reveals that even within the immunosuppressive hepatic microenvironment, protective immune populations can persist and exert clinically meaningful antitumor effects. The liver constitutes a uniquely tolerogenic organ, physiologically primed to prevent excessive immune activation against gut-derived antigens. This baseline immunosuppressive environment may facilitate metastatic colonization by dampening cytotoxic immune responses. In contrast, lung metastases often display distinct immune infiltrates characterized by differential recruitment of dendritic cells and effector lymphocytes, with increased CD4+ T cells and M2-like macrophages compared to primary tumors, suggesting that organ-specific immune niches impose divergent selective pressures on tumor clones [132]. The predominance of CMS4 tumors among lung metastases aligns with the mesenchymal phenotype’s capacity to interact with the lung’s extracellular matrix and stromal elements. Peritoneal metastases exhibit the most striking enrichment of immunosuppressive elements, with increased M2-like macrophages and CAFs compared to both primary tumors and other metastatic sites [128]. This configuration likely reflects the unique challenges of the peritoneal environment, where tumor cells must survive as suspended aggregates, evade mesothelial defenses and establish vascular supply within a stroma-rich, often hypoxic niche. The prominence of M2 macrophages in peritoneal metastases is particularly significant, as these cells promote angiogenesis, tissue remodeling and suppression of adaptive immunity—all features that facilitate peritoneal colonization and progression [124,132]. Thus, these data indicate that metastasis involves progressive immune sculpting. Tumor clones that survive dissemination are those capable of either avoiding immune detection or actively reshaping the immune microenvironment toward tolerance. The result is a metastatic niche that is frequently less inflamed and more resistant to immunotherapeutic intervention than the primary tumor.

### 7.2. MSI-H/dMMR Metastases: A Special Case

Mismatch repair-deficient (MSI-H) CRC represents a distinct immunologic category. Their high tumor mutational burden generates abundant neoantigens, and primary MSI-H tumors are typically heavily infiltrated by cytotoxic lymphocytes with high checkpoint molecule expression [127,133]. This biology underlies their remarkable sensitivity to immune checkpoint blockade [134]. In the metastatic setting, MSI-H tumors account for only a small fraction of cases, but their immunogenic features are largely preserved. The hypermutator phenotype persists after dissemination. Truncal frameshift mutations remain detectable in metastases, and mismatch repair deficiency continues to generate new mutations over time [135]. In theory, this should sustain immune visibility. In practice, however, strong immune pressure drives selection for escape mechanisms. Two alterations are particularly relevant. Loss-of-function mutations in B2M disrupt MHC class I surface expression, impairing presentation of neoantigens to CD8^+^ T cells [127,135]. Similarly, JAK1 or JAK2 loss-of-function mutations render tumor cells unresponsive to interferon-γ signaling, preventing upregulation of antigen presentation machinery [129,135]. A paradox emerges in MSI-H metastases: high neoantigen burden persists, yet immune infiltration may be attenuated or functionally impaired [127,136]. This pressure to evolve likely contributes to heterogeneity in response to checkpoint inhibitors. Site matters here as well. MSI-H tumors with liver metastases appear less responsive to immunotherapy than those with metastases elsewhere [136]. The tolerogenic nature of the liver—constant exposure to dietary and microbial antigens, abundance of suppressive cell populations—may blunt even robust neoantigen-driven immunity [129,136,137]. The mechanisms underlying liver-specific immunotherapy resistance in MSI-H CRC likely mirror those described for microsatellite-stable tumors but are superimposed on a background of high neoantigen availability. TGF-β-mediated T-cell exclusion, TAM-induced immunosuppression and regulatory T-cell accumulation may be particularly pronounced in the hepatic microenvironment, effectively raising the bar for achieving clinically meaningful T-cell responses even when abundant tumor antigens are present [82,127]. This observation has profound clinical implications, suggesting that patients with MSI-H CRC and liver metastases may require combination strategies that target both the tumor (via checkpoint blockade) and the immunosuppressive liver microenvironment (via TGF-β inhibition, TAM depletion, or other approaches) [82,129]. Importantly, MSI-H metastases demonstrate that high mutational load alone is insufficient to guarantee durable immune control. The integrity of antigen presentation machinery and interferon signaling pathways is equally critical. From an evolutionary perspective, MSI-H metastatic progression exemplifies how strong immune pressure accelerates selection for escape mechanisms, reinforcing the principle that metastasis is co-evolutionary—driven by reciprocal adaptation between tumor genomes and host immunity.

### 7.3. The BRAF Paradox: Immunogenic but Suppressed

BRAF V600E–mutant metastases present an intriguing contradiction. They often exhibit higher mutational burden and denser CD8+ T-cell infiltration than other MSS tumors [10,16]. PD-L1 expression is frequently elevated on both tumor cells and macrophages [127,129]. By conventional metrics, they appear immunologically active. Nonetheless, they behave as though immunologically restrained. One explanation lies in the senescence-associated secretory phenotype induced by oncogenic BRAF signaling. Cytokines such as IL-6, IL-8, and TGF-β recruit suppressive myeloid populations and impair cytotoxic T-cell function [124]. Henriques et al. demonstrated that TGF-β-mediated T-cell exclusion is particularly prominent in BRAF-mutant liver metastases, where it establishes a dual barrier—preventing T-cell entry and suppressing those that penetrate the tumor microenvironment [82]. The result is a tumor that is “hot” in appearance but functionally suppressed. This has therapeutic implications. Single-agent checkpoint blockade has shown limited efficacy in BRAF-mutant microsatellite-stable disease, despite its immunogenic features. Combination strategies targeting both MAPK signaling and immune checkpoints are therefore being actively explored [127,129].

## 8. Clinical Translation and Therapeutic Implications

### 8.1. Prognostic and Predictive Genomic Biomarkers

Traditional staging systems rely on anatomical spread, even though phylogenetic evidence indicates that dissemination may precede clinical detection by years [6,24]. Hu et al.’s phylogenetic analyses showed that in the majority of evaluable patients, metastatic seeding appeared to occur when the primary tumor was still microscopically small, in some cases estimated at less than 0.01 cm^3^ in volume [6]. Thus, prognostication must evolve from morphology-based risk assessment toward genomic prediction of metastatic competence. *SMAD4* loss, for example, has been associated with increased metastatic propensity and inferior survival across multiple cohorts [73,74,82]. Mechanistically, disruption of SMAD4 alters TGF-β signaling, diminishes growth restraint, and promotes epithelial plasticity [75]. When this alteration coexists with chromosomal instability signatures and stromal activation programs, it may define tumors that are biologically configured for early dissemination—even if anatomically localized at presentation [11]. Similarly, the CMS4 mesenchymal subtype, characterized by TGF-β activation and stromal infiltration, consistently carries a worse prognosis and higher likelihood of distant relapse [35]. These observations suggest that genomic and transcriptional profiling could refine prognostication beyond traditional stage categories. Liquid biopsy has accelerated this transition. CtDNA offers a composite snapshot of clonal diversity across metastatic sites, mitigating the sampling bias inherent to single-lesion biopsy [35,36]. In practice, plasma profiling often reveals alterations not detected in archival primary tissue. In one study of 84 patients receiving first-line systemic therapy, sequencing of ctDNA alongside tissue DNA uncovered additional mutations in nearly half of cases [36]. Post-treatment ctDNA further revealed new pathogenic alterations that were absent at baseline, underscoring the dynamic nature of clonal evolution under therapeutic pressure. Beyond qualitative mutation detection, quantitative ctDNA metrics appear prognostically meaningful. Higher variant allele frequencies at baseline—particularly for *APC*, *TP53*, and *KRAS*—have been associated with inferior overall survival [36]. These measures likely reflect both tumor burden and biological aggressiveness. Perhaps most important has been the application of ctDNA in MRD detection. In the TRACC Part B study, postoperative ctDNA positivity identified patients at markedly increased risk of recurrence, with ctDNA detection preceding radiographic relapse by a median of more than seven months [35]. Such lead time is not a hypothesis give that it creates a window in which therapeutic intervention could theoretically intercept relapse before clinical manifestation.

### 8.2. Novel Therapeutic Strategies Informed by Genomics

Genomic dissection of metastatic disease suggests that only a minority of tumor cells possess true metastasis-initiating capacity. These cells often display stem-like features, plasticity, and resistance to stress. Markers such as CD26, CD110, and LGR5 have been associated with enhanced tumor-initiating and metastatic potential [124]. Targeting such populations represents a conceptual departure from conventional cytoreduction. The goal shifts from shrinking visible disease to disabling the biological machinery of dissemination. Antibody–drug conjugates, bispecific T-cell engagers, and inhibitors of stemness-associated pathways are being explored with this rationale in mind. Whether selective eradication of metastasis-initiating cells will meaningfully alter clinical trajectories remains to be proven, but the biological premise is compelling [124]. Genomics has also reinforced the idea that metastasis is sustained through reciprocal interaction with organ-specific microenvironments. The liver provides a particularly instructive example. Paracrine HGF–MET signaling from hepatic stroma can blunt the efficacy of EGFR inhibition, even in tumors without MET amplification [73,82]. Preclinical work has shown that HGF-expressing fibroblasts can induce resistance phenotypes in colorectal cancer cells—effects attenuated by HGF neutralization [96]. Additional data suggest that HGF-mediated metabolic rewiring supports tumor survival under nutrient stress [98]. Concomitant targeting of GLUT1 and HGF potently suppressed tumor growth and dissemination, suggesting a rational combination strategy for overcoming microenvironmental resistance [138]. Combination strategies incorporating MET inhibitors alongside tumor-directed therapy aim to dismantle this protective microenvironmental circuitry. Similarly, TGF-β pathway blockade is being explored to counteract immunosuppressive and pro-invasive signaling enriched in metastatic clones with SMAD4 alterations. Strategies aimed at depleting immunosuppressive myeloid populations—via CSF1R inhibition, CTSC targeting, or other approaches—seek to dismantle the stromal infrastructure that protects metastatic clones [86,130]. Kang et al. explored dual blockade of TNFR2 and CD47 to reshape the tumor immune microenvironment and improve antitumor effects in CRC [139]. Such approaches recognize that metastasis is sustained not only by genomic alterations within tumor cells but also by reciprocal signaling with stromal compartments.

MRD-Directed Therapy: Perhaps the most immediate clinical application of evolutionary genomics lies in MRD-guided therapy. The detection of postoperative ctDNA redefines the adjuvant setting: treatment decisions can be based on molecular evidence of residual disease rather than stage alone. The DYNAMIC trial demonstrated that ctDNA-guided management in stage II disease reduced chemotherapy use without compromising recurrence-free survival [140,141]. This represents more than a technical refinement. It signals a shift from empiric risk-based therapy to evolution-informed interception of relapse. If validated broadly, MRD-guided strategies may become central to early-stage management. The BEACON CRC trial established encorafenib (BRAF inhibitor) plus cetuximab (EGFR inhibitor) as standard of care for previously treated BRAF V600E-mutant mCRC, demonstrating improved overall survival compared to chemotherapy (median 8.4 vs. 5.4 months; HR 0.60) [25]. Importantly, the triplet combination including binimetinib (MEK inhibitor) did not improve survival over the doublet, establishing the doublet regimen as standard [25]. The unique biology of BRAF-mutant metastases informs rational combination strategies: the high frequency of peritoneal involvement suggests potential for intraperitoneal delivery approaches; the TGF-β-driven immune suppression in liver metastases provides rationale for combining BRAF/EGFR inhibition with TGF-β blockade [82]; and the immunologically “hot but suppressed” phenotype supports investigation of immunotherapy combinations [127,129]. The CIRCULATE trials (NCT04120701) are evaluating ctDNA-directed escalation or de-escalation strategies across stage II and III disease, with treatment intensification (FOLFOXIRI) for ctDNA-positive patients and de-escalation (observation or reduced therapy) for ctDNA-negative patients [142].

### 8.3. Evolving Diagnostic Standards

As metastatic disease evolves under therapy, the genomic landscape can diverge substantially from the primary tumor. Acquired *RAS* mutations, *HER2* amplification, or MET activation may not be detectable in archival tissue. Case-level analyses have illustrated profound intra-patient heterogeneity, including distinct mutational profiles across synchronous primaries and selective clonal inheritance by metastases [27]. Interestingly, the ERBB4 mutation present in the ancestral primary subclone was undetectable in the resulting metastasis, illustrating both clonal selection during dissemination and the potential for discordance between primary tumor genotype and metastatic therapeutic targets. Wei and colleagues’ demonstration that metastatic tumors inherit multiple genetically distinct subclones from primary tumors—providing evidence for polyclonal seeding—further underscores the limitations of single-lesion biopsy [24]. Different metastatic lesions within the same patient may harbor distinct driver alterations and resistance mechanisms, as Pietrantonio and colleagues documented in the context of anti-EGFR resistance [86]. This inter-lesion heterogeneity means that a single metastasis biopsy may not fully represent the genomic landscape of disseminated disease. Re-biopsy of metastatic lesions, when clinically feasible, provides updated genomic information reflecting current clonal architecture. However, repeated invasive procedures are not always practical.

Comprehensive Plasma Profiling: Comprehensive plasma profiling offers a scalable alternative. Moving beyond single-gene ctDNA assays, emerging approaches employ whole-exome sequencing (WES) or large targeted panels applied to plasma DNA. Such methods capture global mutational burden, structural variants, copy number alterations and resistance mutations simultaneously. Integration of plasma WES with computational clonal deconvolution enables reconstruction of phylogenetic relationships across metastatic sites without direct tissue sampling. Comprehensive profiling offers several advantages over targeted approaches. First, it can detect resistance mechanisms not covered by limited gene panels, including *EGFR* ectodomain mutations, *HER2* amplifications, MET amplifications and fusion events. Second, it enables assessment of genome-wide features such as TMB, MSI status and mutational signatures that may inform immunotherapy selection [96]. Third, longitudinal comprehensive profiling can track clonal dynamics and emerging resistance mechanisms with greater resolution than targeted assays. The proliferation of commercially available and laboratory-developed MRD assays employing divergent methodologies, target selections, bioinformatic pipelines and performance characteristics risks producing inconsistent results that may not solely reflect biological or clinical differences but may instead be consequences of preferential use of particular products in different countries and studies. Standardization and harmonization of ctDNA assays—including consensus definitions of positivity thresholds, analytical validation requirements and clinical utility demonstration—will be essential to ensure that comprehensive plasma profiling delivers reliable and clinically actionable outcomes for patients. As sequencing depth and error-correction technologies improve, plasma-based comprehensive profiling may become the standard for longitudinal monitoring of metastatic CRC. The evolution of diagnostic standards reflects a broader conceptual shift: metastatic CRC is not a static disease defined by initial pathology but a dynamic, evolving genomic ecosystem. Precision oncology in this context demands continuous molecular surveillance, adaptive therapeutic strategies and integration of tumor-intrinsic and microenvironmental genomic data. The ultimate goal is not only to react to resistance but also to anticipate and preempt it, transforming the metastatic odyssey into a controllable evolutionary trajectory rather than an inexorable progression.

## 9. Future Frontiers and Unanswered Questions

### 9.1. Key Research Questions

What are the non-genomic drivers of organotropism? An unresolved question concerns the relative contribution of non-genomic determinants to organotropism. While truncal alterations in genes such as APC, TP53 and KRAS establish a permissive evolutionary substrate, these lesions alone do not explain why certain clones preferentially colonize the liver, lung, or peritoneum [73,84]. Increasing evidence implicates tumor-derived exosomes, secreted metabolites and cytokine gradients in preconditioning distant tissues into receptive “premetastatic niches” [124]. Exosomal integrin profiles, for example, have been proposed to encode organ-specific homing signals, while metabolic crosstalk may prime stromal cells toward a protumorigenic phenotype. Duan et al. reviewed the metabolic crosstalk between colorectal cancer stem cells and their microenvironment, highlighting how metabolic flexibility enables survival in diverse metastatic niches [124]. Liver metastases must adapt to the hepatic metabolic milieu characterized by fluctuating oxygen tension and nutrient gradients, while peritoneal metastases face hypoxic, nutrient-restricted conditions. The metabolic programs selected during organ-specific adaptation—upregulation of *BCAT1*, *ALDH1L2* and glycolytic enzymes—may represent both vulnerabilities and therapeutic opportunities. Deciphering these non-genetic vectors of dissemination will require integrated proteomic, metabolomic and transcriptomic analyses layered onto genomic frameworks. Can we target the metastatic niche preemptively? Closely related is the question of whether the metastatic niche can be therapeutically targeted before overt colonization occurs. The concept of preemptive niche disruption—through modulation of stromal activation, extracellular matrix remodeling, or immune conditioning—represents an attractive but largely untested paradigm in CRC. If micrometastatic colonization depends on defined stromal or vascular states, then pharmacologic interruption of these permissive environments may delay or prevent outgrowth, effectively shifting intervention upstream in the metastatic cascade [96,103,109]. Preclinical evidence supports the feasibility of this concept: TGF-β inhibitors disrupt the immunosuppressive liver microenvironment and enhance immunotherapy efficacy [103,131]; HGF/MET blockade dismantles paracrine protective circuits [96,98]; and CSF1R inhibitors deplete tumor-associated macrophages that support metastatic growth [109]. Clinical translation of niche-directed prevention will require identifying patients at highest risk of metastatic relapse—those with detectable MRD post-resection—and intervening with microenvironment-targeted agents before radiographic progression. The lead time of 6–11 months between ctDNA detection and clinical relapse [109,110] provides a window for such preemptive strategies. Future trials will need to test whether combining conventional chemotherapy or targeted therapy with niche-modulating agents can prevent or delay metastatic outgrowth in MRD-positive patients. What defines the dormancy-to-outgrowth switch genomically? An interesting biological transition in metastatic evolution is the dormancy-to-outgrowth switch. Disseminated tumor cells (DTCs) can persist in a quiescent state for years or decades before reactivation, a phenomenon responsible for late relapses long after primary tumor resection [109,124]. While transcriptional programs involving NR2F1, p38 MAPK signaling and cell-cycle repression have been implicated in dormancy maintenance, the precise genomic and epigenomic triggers that license proliferative escape remain poorly defined [109]. The balance between p38 and ERK signaling appears critical: high p38/low ERK activity favors dormancy, while the inverse promotes proliferation. Is reactivation driven by acquisition of new mutations, epigenetic reprogramming, microenvironmental perturbations, or stochastic clonal selection? Do dormant cells accumulate somatic mutations during quiescence, eventually acquiring drivers that enable outgrowth? Or is reactivation purely epigenetic and microenvironmentally triggered, with outgrowth occurring without new genomic alterations? Longitudinal sampling and integrative modeling of minimal residual disease will be essential to answer this question, as it lies at the heart of late recurrence. Single-cell sequencing of dormant DTCs and matched overt metastases from the same patients will be required to resolve this fundamental question.

### 9.2. Technological Horizons

Single-Cell Multi-Omics of Circulating Tumor Cells (CTCs) and DTCs: The metastatic bottleneck imposes stringent selection that reduces genetic diversity, but the rare cells that successfully complete all steps of the cascade possess unique phenotypic attributes that bulk sequencing cannot resolve. Single-cell multi-omic technologies now enable profiling of CTCs and DTCs at unprecedented resolution, linking genotype to phenotype at the level of individual metastatic precursors [124]. By simultaneously interrogating DNA mutations, chromatin accessibility, RNA expression and protein signaling states, investigators can define the functional programs that distinguish metastasis-initiating cells from the broader tumor population. Advances in microfluidic capture and low-input sequencing allow simultaneous assessment of genomic copy number, mutational status and transcriptomic programs from individual CTCs. That integrative resolution is critical for understanding plasticity, lineage transitions and therapy resistance in disseminating clones. Metastatic colonization occurs at dynamic tumor–stroma interfaces where cancer cells engage hepatocytes, alveolar epithelium, mesothelial layers, endothelial cells and immune infiltrates. Bulk sequencing obscures these localized interactions. Spatially resolved transcriptomic and proteomic platforms now enable high-dimensional mapping of cellular neighborhoods, revealing gradients of cytokine signaling, immune exclusion zones and metabolic compartmentalization at single-cell resolution [124]. Applied to metastatic lesions, spatial transcriptomics can reveal how cancer-associated fibroblasts (CAFs) at the invasive front differ from those in the tumor core, how immune cells are organized relative to tumor cells and which ligand-receptor pairs mediate critical crosstalk. Integration of spatial transcriptomics with genomic data from the same lesions can identify whether specific mutations are associated with distinct spatial organizations—for example, whether SMAD4-mutant metastases exhibit different immune exclusion patterns than SMAD4-wild-type lesions. Applying these tools to early metastatic foci may illuminate the cooperative circuits that enable successful colonization.

Longitudinal cfDNA Methylation Tracking: While ctDNA mutation analysis has revolutionized MRD detection and resistance monitoring, its sensitivity is limited by the need to track pre-identified patient-specific mutations. An emerging complementary approach leverages cell-free DNA (cfDNA) methylation patterns for both detection and tissue-of-origin mapping [85,109]. Tumor-derived cfDNA retains cancer-specific methylation marks that distinguish it from DNA shed by normal cells. Genome-wide methylation sequencing can detect the presence of cancer DNA at extremely low fractions—potentially exceeding the sensitivity of mutation-based approaches—and simultaneously infer the tissue of origin based on methylation patterns. Unlike somatic mutations, epigenetic patterns encode lineage identity and cellular state, providing an additional dimension for reconstructing metastatic trajectories. For patients with metastatic CRC of unknown primary or with suspected second primaries, cfDNA methylation could distinguish recurrence from new primary cancers. Longitudinal methylation tracking may also capture epigenetic evolution during treatment, revealing resistance mechanisms that operate without genomic mutation. Serial methylome tracking could therefore enhance early detection and refine our understanding of evolutionary plasticity. Yu and colleagues highlight the potential of integrating advanced imaging with multi-omic cfDNA profiling to create a comprehensive surveillance paradigm [109]. By combining ctDNA mutation analysis for clonal dynamics, cfDNA methylation for sensitive detection and tissue mapping and radiographic imaging for spatial localization, this integrated approach could transform postoperative surveillance and early intervention.

### 9.3. Conceptual Challenges

Defining “Metastasis Driver” versus “Passenger”: One of the most persistent conceptual challenges is distinguishing true “metastasis drivers” from passengers that accumulate during clonal expansion. Many alterations observed in metastatic lesions may be permissive rather than causative—facilitating survival in a new microenvironment without being strictly required for dissemination [79]. A mutation present in all metastases but absent from the primary tumor could be a driver acquired during dissemination, but it could also be a passenger that arose in the metastatic founder clone after it departed the primary but before colonization—selected not for its functional contribution but simply because it hitchhiked with the true driver. The distinction is critical for therapeutic targeting. Alterations that are truly metastatic drivers represent high-priority drug targets; passengers, by contrast, are irrelevant to intervention. Rigorous functional validation, ideally through organ-specific in vivo models and CRISPR-based perturbation screens, is necessary to discriminate essential determinants from evolutionary byproducts. Turajlic and Swanton articulate this challenge in their evolutionary theorem of metastasis, emphasizing that the same genomic alteration may have different functional consequences depending on timing and context [79]. A *TP53* mutation acquired early in tumorigenesis may be a true driver of metastatic progression by enabling genomic instability and apoptosis resistance; the same mutation acquired late in a subclone that has already metastasized may be functionally neutral. The distinction depends not only on the gene but on its evolutionary context. Another underappreciated axis of metastatic evolution is aneuploidy and CIN. Rather than acting through discrete driver mutations alone, CIN may enhance metastatic capacity by increasing evolvability—the ability of tumor populations to explore adaptive fitness landscapes under selective pressure [12,79]. Large-scale copy-number alterations can simultaneously modulate multiple oncogenic pathways, buffer deleterious mutations and generate phenotypic heterogeneity that fuels selection. In this view, metastasis may not depend solely on acquiring specific mutations, but on achieving a genomic state permissive for rapid adaptive diversification. The distinction matters for therapeutic strategy. If CIN directly drives metastasis through specific aneuploidy-associated phenotypes (e.g., immune evasion via cytosolic DNA sensing), then targeting CIN itself could be antimetastatic. Bakhoum and colleagues provided mechanistic insight into how CIN promotes metastasis, demonstrating that chromosomal mis-segregation generates micronuclei whose rupture exposes cytosolic DNA, activating the cGAS-STING pathway and promoting a metastatic gene expression program [12]. It suggests that CIN is more than permissive since it actively contributes to metastatic phenotypes through inflammation-like signaling. If CIN merely increases evolvability, then strategies should focus on constraining evolution—through adaptive therapy, immunotherapy that maintains immune surveillance, or targeting the fittest clones before resistance emerges. The extent to which these mechanisms operate across diverse CRC subtypes and metastatic sites remains to be determined.

## 10. Conclusions

Metastatic CRC is not defined by a single genomic event but by a continuous evolutionary journey. From the establishment of primary tumor heterogeneity to early dissemination, organ-specific adaptation, immune sculpting and therapeutic resistance, each phase imposes selective pressures that shape the genomic landscape of progressing disease. The metastatic bottleneck reduces clonal diversity while enriching for traits that enable dissemination—*SMAD4* loss, *PTEN* inactivation and metabolic reprogramming. Subsequent organ-specific adaptation yields distinct molecular signatures: liver metastases exhibit Wnt hyperactivation and TGF-β-driven immune suppression; peritoneal tumors display mucinous features and epithelial–mesenchymal plasticity; brain metastases show HER2 enrichment. The immune microenvironment evolves toward increasingly immunosuppressive configurations, particularly in the liver, while MSI-H tumors preserve neoantigen load but acquire immune evasion mutations. Therapeutic resistance follows predictable evolutionary trajectories under drug pressure, with metastasis-specific mechanisms including microenvironmental protection and cellular dormancy adding further complexity. The clinical future lies in interception: using liquid biopsies to detect molecular residual disease months before radiographic progression, targeting both tumor-intrinsic vulnerabilities and permissive metastatic niches and adapting therapy dynamically to anticipate rather than merely react to resistance. Understanding the genomic evolution from primary to metastasis is the essential foundation for transforming colorectal cancer from a lethal systemic disease into a controllable chronic condition.

## Figures and Tables

**Table 1:** Evolutionary Models of Metastatic Dissemination in Colorectal Cancer. Synthesis of phylogenetic models derived from multi-region sequencing studies.

Model	Key Characteristics	Genomic Evidence	Clinical Implications	References
Linear Progression	Sequential accumulation of driver mutations; metastasis as late event	Clonal ordering of *APC* → *KRAS* → *TP53* mutations in primary tumors	Late detection limits curative intervention	[3,4]
Early Dissemination	Metastatic seeding while primary tumor remains clinically undetectable (<0.01 cm^3^)	81% of patients show evidence of early seeding; low primary-metastasis genomic divergence	Occult micrometastases at diagnosis; mandates effective systemic therapy	[6,8,41]
Late Dissemination	Metastases arise from dominant late primary tumor clones	Metastatic lineages diverge after substantial subclonal diversification within primary	May be more amenable to local control strategies	[7,42]
Parallel Evolution	Independent subclonal evolution at primary and metastatic sites	Branching phylogenies with private mutations in metastases	Single biopsy insufficient for comprehensive genomic profiling	[5,25]
Polyclonal Dissemination	Multiple genetically distinct clones seed metastases simultaneously or sequentially	Metastatic tumors inherit multiple distinct subclones from primary; different lesions harbor distinct driver alterations	Requires multi-lesion sampling for treatment planning; ctDNA captures composite clonal architecture	[24]
Metastasis-to-Metastasis Seeding	Metastatic clones themselves serve as sources of further dissemination	Phylogenetic reconstruction revealing ovarian metastases arising from peritoneal or lymph node deposits	Explains rapid progression and multi-organ involvement	[41,43]
BRAF-Driven Early Dissemination	*BRAF V600E* mutation as truncal event; early metastatic seeding; distinct organotropism (peritoneal > liver)	*BRAF V600E* present in all metastases; low primary-metastasis divergence; *RNF43* co-mutation enrichment (28–32%)	High risk of peritoneal recurrence; reduced ctDNA shedding complicates monitoring; poor prognosis even in early-stage	[8,41,44,45]

**Table 2:** Organ-Specific Genomic and Immune Features of CRC Metastases.

Metastatic Site	Genomic Signatures	Immune Microenvironment	BRAF V600E Prevalence	BRAF-Specific Features	ctDNA Shedding	Clinical Implications	References
**Liver**	– *APC* mutations (64–76%) – *TP53* mutations (82%) – *SMAD4* inactivation (14–24%) – Wnt pathway hyperactivation – *BCAT1*, *ALDH1L2* upregulation	– “Cold” phenotype – T-cell exclusion via TGF-β – Enriched M2 macrophages – Reduced TCR diversity – Vδ1 T cells with antitumor potential	Low (5–8%)	– TGF-β-mediated suppression prominent when BRAF-mutant – SPP1+ macrophage enrichment – Potential for TGF-β blockade combinations	High (23.1% median VAF) Concordance: 91–96%	– Reduced immunotherapy efficacy – Amenable to HGF/MET and TGF-β targeting	[73,74,82,83]
**Lung**	– *KRAS G12C* enrichment – NKX2-1 (TTF-1) aberrant expression	– Increased CD4+ T cells – M2-like macrophages – Distinct dendritic cell recruitment	Intermediate (10–15%)	– May require different targeted approaches – Less characterized	Low (0.4% median VAF) Concordance: 64–66%	– Requires concurrent tissue/plasma testing – Potential for KRAS G12C inhibitors	[84,85]
**Peritoneal**	– *KRAS* mutation prevalence – *MUC2*, *MUC5AC* upregulation – *CDH1* loss (EMT)	– Marked M2 macrophage enrichment – Increased CAF infiltration – Immunosuppressive myeloid populations	Highest (35–46%)	– Mucinous histology enrichment – SASP-driven inflammation – Reduced ctDNA shedding (median VAF 0.4%) – Poor response to targeted therapy	Lowest (0.4% median VAF) Concordance: 56%	– Aggressive clinical phenotype – Tissue biopsy essential for genotyping	[74,84,85]
**Brain**	– HER2 amplification (18%) – *KRAS* mutations (76%) – Lower TMB associated with improved intracranial PFS	– TIL enrichment predicts longer OS – Immune-privileged microenvironment	Limited data (~8–10%)	– Rare in BRAF-mutant series – May co-occur with HER2 amplification	Not systematically evaluated	– Rare (1–3% of CRC) – HER2 targeting opportunities – Context-dependent *KRAS* effects	[59]

## Data Availability

All data generated are available by the leading author upon request.

## Abbreviations

The following abbreviations are used in this manuscript:

mCRC	Metastatic colorectal cancer
MSI-H	Microsatellite instability high
ctDNA	Circulating tumor DNA
CRC	Colorectal cancer
CIN	Chromosomal instability
CMS	Consensus Molecular Subtypes
TME	Tumor microenvironment
MRS	Multi-region sequencing
CAFs	Cancer-associated fibroblasts
MRD	Minimal residual disease
CRC-OM	Ovarian metastases originating from CRC
MET	Mesenchymal-to-epithelial transition
VAF	Variant allele frequency
DTCs	Disseminated tumor cells
CTCs	Circulating Tumor Cells
cfDNA	Cell-free DNA
